# Cascade amplification of tumor chemodynamic therapy and starvation with re-educated TAMs via Fe-MOF based functional nanosystem

**DOI:** 10.1186/s12951-023-01878-3

**Published:** 2023-04-11

**Authors:** Xinmin Zheng, Xiang Li, Siyu Meng, Guolin Shi, Hui Li, Huiping Du, Liangliang Dai, Hui Yang

**Affiliations:** 1 School of Life Sciences, Northwestern Polytechnical University, Xi’an, 710072 China; 2 Institute of Medical Research, Northwestern Polytechnical University, Xi’an, 710072 China

**Keywords:** Cascade amplification, Tumor starvation, Chemodynamic therapy, Re-education macrophages, Fe-Mof nanosystem

## Abstract

Tumor microenvironment is characterized by the high concentration of reactive oxygen species (ROS), which is an effective key used to open the Pandora’s Box against cancer. Herein, a tumor-targeted nanosystem HFNP@GOX@PFC composed of ROS-cleaved Fe-based metal–organic framework, hyaluronic acid (HA), glucose oxidase (GOX) and perfluorohexane (PFC) has been developed for tumor cascade amplified starvation and chemodynamic therapy (CDT). In response to the high concentration of hydrogen peroxide (H_2_O_2_) intratumorally, HFNP@GOX@PFC endocytosed by tumor cells can specially be disassembled and release GOX, PFC and Fe^2+^, which can collectively starve tumor and self-produce additional H_2_O_2_ via competitively glucose catalyzing, supply oxygen to continuous support GOX-mediated starvation therapy, initiate CDT and cascade amplify oxidative stress via Fe^2+^-mediated Fenton reaction, leading to the serious tumor damage with activated p53 signal pathway. Moreover, HFNP@GOX@PFC also significantly initiates antitumor immune response via re-educating tumor-associated macrophages (TAMs) by activating NF-κB and MAPK signal pathways. In vitro and in vivo results collectively demonstrate that nanosystem not only continuously initiates starvation therapy, but also pronouncedly cascade-amplify CDT and polarize TAMs, consequently efficiently inhibiting tumor growth with good biosafety. The functional nanosystem combined the cascade amplification of starvation and CDT provides a new nanoplatform for tumor therapy.

**Supplementary Information:**

The online version contains supplementary material available at 10.1186/s12951-023-01878-3.

## Introduction

Chemodynamic therapy (CDT) driven by Fenton/Fenton-like reaction is known as a potential tumor therapeutic modality with minimal side effects [1, 2]. Generally, nano-catalysts and H_2_O_2_ are the two key initiators and limitation factors of CDT [3, 4]. Benefiting from the high catalytic activity, iron (Fe)-based nanoparticles have been frequently used to construct CDT platform against tumors [5]. However, the inadequate and limited content of endogenous H_2_O_2_ in tumors seriously restricts the efficiency of CDT with the unsatisfactory therapy effect, owing to the tumor heterogeneity. Besides, the overexpressed reducing substances in tumor microenvironment, such as glutathione, can counteract the production of hydroxyl radical (·OH) and extra reduce the CDT therapeutic efficacy [6]. Therefore, it is necessary to fabricate a functional Fe-based nanosystem integrating with H_2_O_2_ self-supply capacity for improved tumor CDT efficiency. For one thing, the excess H_2_O_2_ generated by nanosystem converts the reductive property of glutathione and cascade amplifies CDT efficiency [7]. For another thing, the excess ROS also possesses the initiation antitumor immune response potential via re-educating tumor-associated macrophages (TAMs) [8], devoting to effective tumor killing.

Typically, glucose oxidase (GOX) seems to be a good choice to solve the shortage of endogenous H_2_O_2_. GOX is an endogenous oxidoreductase, which can effectively catalyze glucose to gluconic acid and generate abundant H_2_O_2_ [9]. Notably, glucose, as a catalytic substrate for GOX and nutritional supplier, plays a vital role in tumor growth. Since the proliferation of tumor cells mainly depends on aerobic glycolysis, which makes tumor cells more sensitive to the change in glucose concentration [10]. It is reported that cancer cells need to burn almost 20-fold glucose to get the same amount of energy compared with normal cells [11]. Therefore, the GOX combined with the CDT strategy can not only provide enough endogenous H_2_O_2_ for cascade amplifying CDT efficiency, but also significantly consume glucose for tumor starvation therapy, displaying the potential antitumor effect [10, 12, 13]. However, the catalysis activity of GOX is severely limited by the relatively low delivery efficiency and hypoxia in vivo, the significant characteristics of tumor microenvironment. Thus, efforts to improve the delivery efficiency of GOX and self-supply exogenous O_2_ simultaneously are critical for the combined tumor CDT and GOX-based starvation therapy.

Metal–organic frameworks (MOFs) as applicable nanocarriers have been frequently reported for drug delivery with superior delivery efficiency, resulting from the natural advantages of adjustable pores, high surface area, diverse structure and easy functionalization [14, 15], which could protect the biologically active drugs (e.g., enzyme, GOX, etc.) from inactivation caused by the external environment, so as to deliver drugs to tumors with high efficiency and good safety. Inspired by the high Fenton reaction activity of Fe-based nanoparticles and excellent drug delivery capacity, Fe-based MOF is thus recommended as a class of natural CDT initiators and GOX carriers. Considering the perfluorohexane (PFC) has been extensively evaluated as a substitute oxygen carrier, it could effectively self-supply exogenous O_2_ to overcome above mentioned hypoxia and limitation obstacles of GOX-mediated starvation therapy. Hence, Fe-based MOF co-loaded with GOX and PFC may serve as a promising platform able to self-supply exogenous O_2_ and H_2_O_2_, which could effectively cascade amplify tumor CDT and starvation therapy efficiency with potential TAMs regulation, realizing the desired cancer damage. However, poor-degradability, off-targeting capacity and associated potential biosafety have always restricted the application of the Fe-based MOF system. Therefore, a new solution to improve the biocompatible and active targeting of Fe-MOFs for tumor CDT and starvation therapy is urgently needed.

**Fig. 1:**
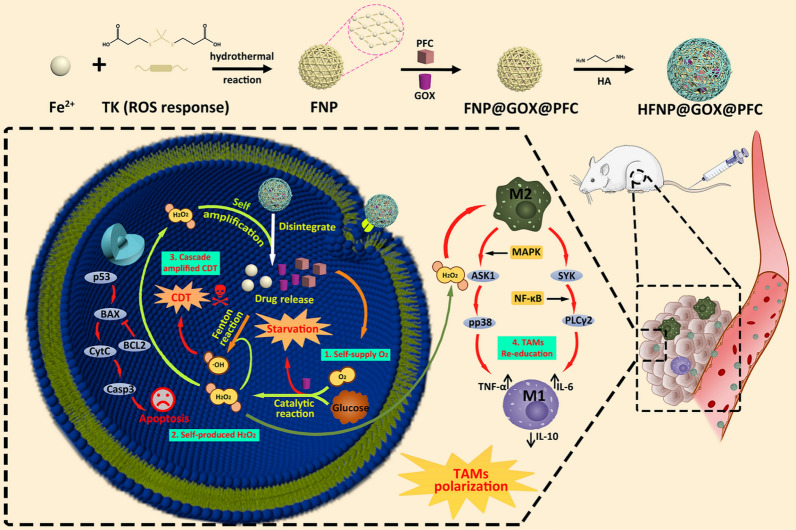
Synthesis routes and schematic illustration of HA-modified ROS-responsive HFNP@GOX@PFC nanosystem for combined CDT, starvation and re-educated TAMs therapy

Based on the above considerations, we herein developed a tumor-targeted functional nanosystem based on ROS-responsive degradable Fe-MOF to co-loaded GOX & PFC for the cascade amplified CDT and starvation therapy with TAMs polarization. The ROS-responsive degradable Fe-MOF was first synthesized through hydrothermal reaction composed of 2,2'-[propane-2,2-diylbis(thio)]diacetic acid as ROS-sensitive agent thioketal (TK) and Fe^2+^ ion. Subsequently, GOX as exogenous H_2_O_2_ supplier and starvation inducer and PFC as O_2_ provider are co-loaded into Fe-MOF. Finally, the tumor targeting molecular hyaluronic acid (HA) is introduced into the surface of drug loaded Fe-MOF, harvesting the resultant functional nanosystem (HFNP@GOX@PFC). After it is injected into the venous system, the HA-functionalized Fe-MOF nanosystem could prolong circulation time and accumulate in the tumor site through passive targeting of the enhanced permeability and retention effect (EPR) and active targeting of HA receptor-ligand mediated endocytosis. HFNP@GOX@PFC uptake by tumor cells is then disassembled in response to the relatively high concentrations of ROS in the cytoplasm, leading to the release of O_2_ supplier PFC, H_2_O_2_ supplier and starvation inducer GOX, and CDT trigger agent Fe^2+^. Subsequently, plenty of O_2_ self-generated by PFC reduces hypoxia. Moreover, GOX competitively reacts with intracellular glucose and sufficient O_2_ generated by PFC to starve tumor and produce abundant H_2_O_2_, which not only accelerates the disassembly of HFNP@GOX@PFC and drug release, but also promotes the re-education of TAMs and provides the fuel for CDT. The released Fe^2+^ reacts with the fuel of H_2_O_2_ self-supplied by GOX to cascade amplify CDT and induce effective tumor damage. The above continuous “Self-Amplified” process can proceed continuously in the tumor microenvironment to boost tumor killing efficacy with activated p53 signal pathway through combining starvation and CDT. More importantly, ROS generated by above cycle could additionally boost tumor immune response via re-educating TAMs by activating NF-κB and mitogen-activated protein kinase (MAPK) signal pathways. Eventually, the multiple therapies induce the superior tumor therapy effect (Fig. 1). Hence, we hypothesized that the functional nanosystem HFNP@GOX@PFC could achieve desired antitumor therapy by combining starvation and CDT with TAMs re-education.

## Results and discussion

### Preparation and characterization of HFNP@GOX@PFC nanosystem

**Fig. 2:**
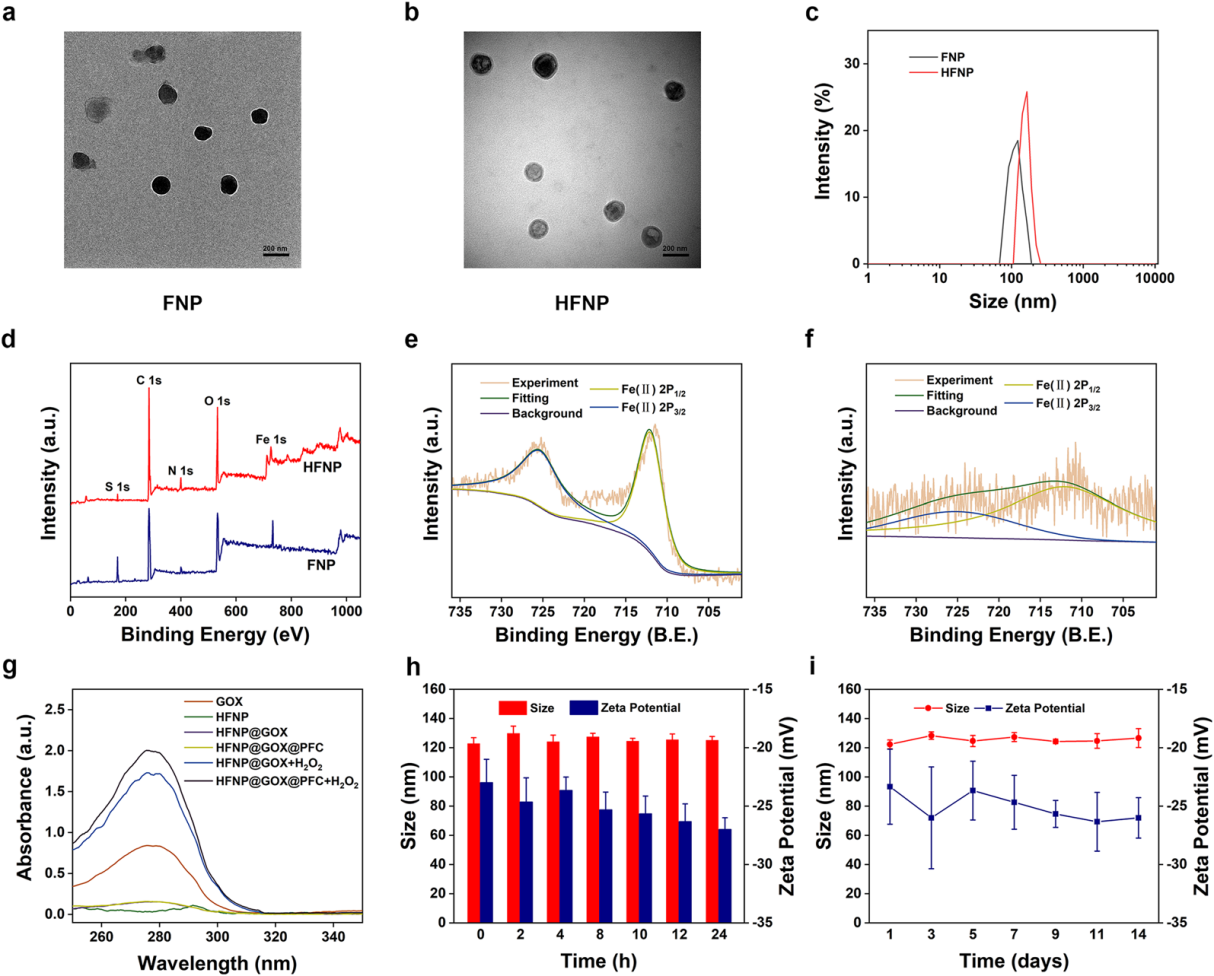
**a** TEM images of FNP and **b** HFNP. **c** DLS dates of FNP and HFNP. **d** XPS spectra survey of FNP and HFNP. XPS spectra of Fe 2p oribit for **e** FNP and **f** HFNP. **g** The absorption spectra of GOX, HFNP, HFNP@GOX with or without H_2_O_2_, and HFNP@GOX@PFC with or without H_2_O_2_. **h** Short-term stability and **i** long-term stability of HFNP@GOX@PFC. Scale bar: 200 nm. Error bars present as mean ± SD (n = 6)

The degradable Fe-Mof nanoparticles (FNP) were synthesized by conjugation Fe^2+^ with carboxyl groups from ROS-cleaved linker TK using hydrothermal reaction. In order to improve biocompatibility and tumor targeting capacity, HA coat-layer was introduced into the surface of FNP nanoparticles, which was used to prolong the circulation in blood vessels and impart tumor targeting feature to HFNP nanosystem. Transmission electron microscopy (TEM), dynamic light scattering (DLS), zeta potential measurement, X-ray diffraction (XRD), Fourier Transform Infrared Spectrometer (FTIR), X-ray photoelectron spectroscopy (XPS), nuclear magnetic resonance (NMR), ultraviolet and visible spectrophotometry (UV–vis) were employed to monitor the functionalization of HFNP nanosystem. Firstly, both FNP and HFNP exhibited the distinctively spherical morphology with good mono-dispersity detected by TEM (Fig. 2a, b), and the uniform sizes were respectively distributed at 130 ± 9.2 and 158 ± 6.5 nm, which was consistent with the corresponding DLS results (Fig. 2c). As companying with the introduction of HA, the size of HFNP displayed an obvious increase, indicating the successful modification of HA. Simultaneously, the zeta potential of HFNP was also significantly reduced from − 22 ± 1 to -25 ± 0.4 mV after the conjugation of HA (p < 0.01, Additional file 1: Fig. S1), suggesting the successful construction of HFNP nanosystem again. Secondly, XRD results showed that the obvious peaks of 5.58°, 8.62° and 10.1° were observed in FNP sample (Additional file 1: Fig. S2), attributed to the typical structure of Fe-Mof nanoparticles [16]. After conjugation with HA molecule, similar peaks of 5.62°, 8.64° and 10.08° were also displayed in HFNP sample, indicating again the synthesis of FNP. More importantly, the new peak of 25.18° was shown in the XRD spectrum of HFNP, it was ascribed to the characteristic peak of HA, which was consistent with previous research [17]. The result further confirmed the successful modification of HA and fabrication of HFNP. Thirdly, XPS was carried to analyze the elements of FNP and HFNP. As shown in Fig. 2d, four typical peaks of C, N, Fe, O and S elements were observed in both FNP and HFNP, indicating the successful synthesis of FNP and the introduction of HA in HFNP [18]. Moreover, the Fe in FNP (Fig. 2e) and HFNP (Fig. 2f) nanocarriers were confirmed to be ferrous ions as indicated by the two strong binding energy peaks appeared at 712 and 726 eV, responsible for the appearances of Fe 2p3/2 and Fe 2p1/2 [19]. Taken together, those results indicated the successful formation of HFNP nanosystem. Additionally, the typical characteristic peaks of HA (ranged from 3.12 to 3.85 ppm and 1.9 ppm) displayed in the NMR spectrum of HFNP (Additional file 1: Fig. S3), which was attributed to the signals of the protons in the sugar rings and methyl protons of the N-acetyl group of HA, confirming again the successful introduction of HA on the surface of FNP [20, 21]. Finally, the FTIR also revealed the conjugation of HA, revealed by the typical characteristic peaks of various functional groups (Additional file 1: Fig. S4). The results collectively suggested that the ROS-responsive degradable Fe-Mof nanosystem with HA modification was successfully constructed.

Subsequently, the drug loading content and drug loading efficiency of GOX and PFC in HFNP@GOX@PFC nanosystem were detected via UV–vis spectrophotometry and gas chromatography (GC). As shown in Fig. 2g, both HFNP@GOX and HFNP@GOX@PFC groups without H_2_O_2_ treatment had no obvious GOX release, proved by the negligible absorption intensity of GOX at 276 nm, indicating the successful GOX loading in the nanosystem. Furthermore, the loading content and encapsulation efficiency of GOX in HFNP were calculated as 2.42% and 82.5% based on the associating standard curve (Additional file 1: Fig. S5). Moreover, after treatment with H_2_O_2_, both HFNP@GOX and HFNP@GOX@PFC groups showed the typical GOX absorption peak at 276 nm compared with GOX group, implying the effective drug release. Furthermore, the loading content and efficiency of PFC in HFNP@GOX@PFC nanosystem were measured with GC. HFNP@GOX@PFC exhibited the obvious elution peak of PFC at 1.152 min the same as PFC (Additional file 1: Fig. S6), indicating the successful encapsulation of PFC. The loading content and encapsulation efficiency of PFC in HFNP@GOX@PFC were determined as 32.37% and 78.07%, according to the corresponding standard curve (Additional file 1: Fig. S7). These results indicated that the nanosystem had been fabricated successfully. Additionally, the stability of HFNP@GOX@PFC nanosystem was evaluated. As shown in Fig. 2h, i, the size & zeta potential of HFNP@GOX@PFC had no significant change, despite the short-term (0–24 h) in 10% fetal bovine serum of PBS or long-term (1–14 day) in PBS incubation, proving that HFNP@GOX@PFC had great biostability & storage stability, and it was conducive to the drug delivery as nanocarriers for tumor therapy in vivo and in vitro.

**Fig. 3:**
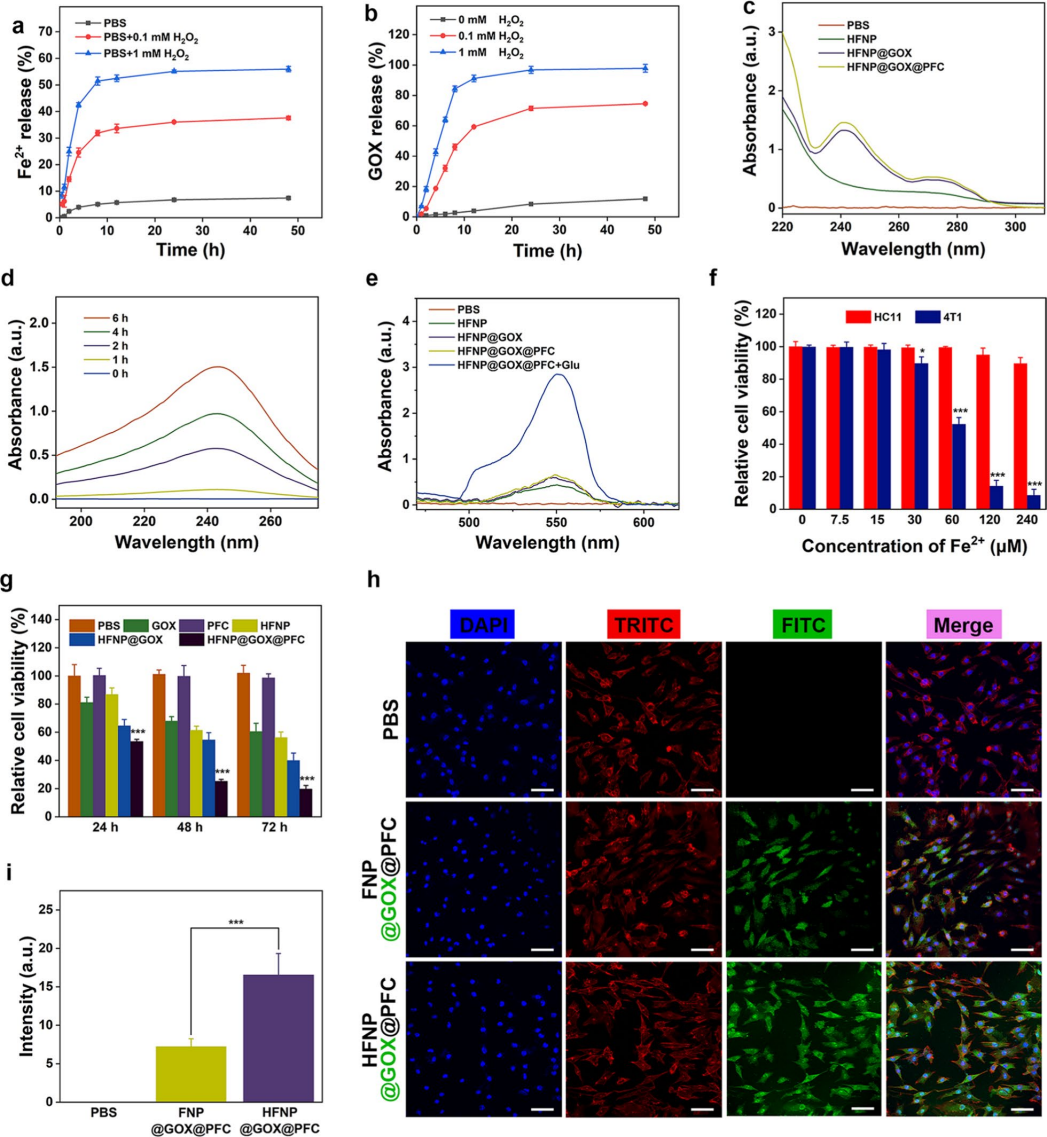
Cumulative release of **a** Fe^2+^ and **b** GOX from HFNP@GOX@PFC after treatments with different concentrations of H_2_O_2_. **c** The self-produced H_2_O_2_ capability of HFNP@GOX@PFC nanosystem. **d** Time-dependent accumulation of H_2_O_2_ of HFNP@GOX@PFC after treatments with glucose for different times. **e** The self-amplificated generation of ·OH of PBS, HFNP, HFNP@GOX and HFNP@GOX@PFC with or without glucose. **f** Cytotoxicity of HC11 and 4T1 cells after treatment with HFNP@GOX@PFC at different doses of Fe^2+^ for 24 h. **g** Time-dependent cytotoxicity of 4T1 cells treated with PBS, GOX, PFC, HFNP, HFNP@GOX and HFNP@GOX@PFC for 24 h, 48 h and 72 h. **h** CLSM images and **i** fluorescence intensity of 4T1 cells after treated with PBS, FNP@GOX@PFC and HFNP@GOX@PFC for 12 h. Scale bar: 200 μm. Error bars present as mean ± SD (n = 6)

The vital features of HFNP@GOX@PFC nanosystem are ROS-responsive Fe-MOF disassembly, drug release, self-production of H_2_O_2_ and cascade amplification of CDT. Firstly, TEM, DLS and UV–vis spectrophotometry were used to monitor the ROS-responsive Fe-MOF disassembly & drug release traits. As demonstrated in Additional file 1: Fig. S8, the initial spherical structure was obviously changed to collapse structure when exposed to H_2_O_2_ (Fig. 2a, b *vs*. Additional file 1: Fig. S8), which was consistent with DLS (Additional file 1: Fig. S9), suggesting the ROS-responsive disassembly of FNP and HFNP. The reason could be explained that the ROS-sensitive linker of the TK could actually cleave in response to H_2_O_2_ stimuli. Thus, FNP and HFNP composed of Fe^2+^ ions and TK agent could be rapidly disintegrated triggered by H_2_O_2_, which is beneficial for the following drug release. Furthermore, both the ROS-responsive Fe^2+^ and GOX release behaviors of HFNP@GOX@PFC nanosystem were quantitatively measured using o-phenanthroline method and UV–vis spectrophotometry. H_2_O_2_ was used to simulate the intracellular ROS concentration of tumor cells and analyze the drug release of nanosystem. As shown in Fig. 3a, there was barely any Fe^2+^ release in the control group (PBS) upon 48 h, indicating the good encapsulation, stability and potential biocompatibility of HFNP@GOX@PFC nanosystem (owing to no leakage during the blood circulation). In contrast, the release amount of Fe^2+^ was dramatically increased in the presence of H_2_O_2_, showing the H_2_O_2_ concentration-dependence drug release pattern (Fig. 3a, 0.1 mM H_2_O_2_ *vs*. 1 mM H_2_O_2_), suggesting that HFNP@GOX@PFC nanosystem actually possesses the ROS-responsive drug release capability. At the same time, GOX also showed H_2_O_2_ concentration-dependent release behavior, and the release amount reached 69% and 91% (almost complete drug release) in 0.1 mM H_2_O_2_ and 1 mM H_2_O_2_ treatment groups, while the release amount of GOX was negligible in control group as well (Fig. 3b). The result further confirmed again the effective ROS-responsive drug release features of HFNP@GOX@PFC nanosystem with good stability. Notably, H_2_O_2_ led to the disintegration of HFNP@GOX@PFC nanosystem and the release of Fe^2+^ with H_2_O_2_-dependent pattern, which was benefited for the subsequent CDT treatment.

Secondly, UV–vis spectrophotometry was used to characterize the self-production capability of H_2_O_2_ of HFNP@GOX@PFC nanosystem, since H_2_O_2_ possesses the natural characteristic peak at 240 nm. As shown in Fig. 3c, GOX loaded nanoparticles (HFNP@GOX and HFNP@GOX@PFC) co-incubated with glucose exhibited the obviously characteristic peak of H_2_O_2_ compared to control (PBS) and negative HFNP groups, confirming the self-generation of H_2_O_2_. Simultaneously, HFNP@GOX@PFC nanosystem could generate gluconic acid via GOX-mediated catalytic reaction, as revealed by the reduction of pH value (Additional file 1: Fig. S10). It also indicated that the catalytic activity of GOX was not affected by the encapsulation of the HFNP@GOX@PFC nanosystem. Furthermore, the self-supply H_2_O_2_ capability of the HFNP@GOX@PFC nanosystem displayed a time-dependent pattern (Fig. 3d), and the yield of H_2_O_2_ at 6 h incubation was about 160 times that of 1 h, proving again self-produced H_2_O_2_ feature of HFNP@GOX@PFC nanosystem. Notably, the gluconic acid generated from GOX and H_2_O_2_ actually induced the decomposition of HFNP@GOX@PFC (Additional file 1: Fig. S11). Therefore, HFNP@GOX@PFC nanosystem endowed with self-generation of H_2_O_2_ capacity would not only accelerate nanocarriers disassembly and drug release in turn, but also provide the fuel “H_2_O_2_ and Fe^2+^” for Fenton reaction, leading to the cascade amplification of CDT and desired antitumor effect.

Thirdly, the generation level of ·OH as the direct indicator labelled CDT was investigated via Hydroxyl Free Radical assay Kit for evaluating the cascade amplification of CDT. As shown in Fig. 3e, compared with control (PBS), HFNP, HFNP@GOX and HFNP@GOX@PFC groups co-incubated with H_2_O_2_ all showed the typical ·OH peaks without glucose input, which further proved the successful construction of Fe^2+^-based Mof with good Fenton reaction ability. More importantly, the production of ·OH increased sharply after adding glucose, because the catalytic activity of GOX was amplified by glucose and much more H_2_O_2_ produced, thus amplifying the Fenton reaction of the nanosystem. Considering the good stability, ROS-responsive Fe-Mof disassembly & drug release, self-production of H_2_O_2_ and cascade amplification of CDT performance of the nanosystem, it inspired us to further explore the antitumor effect in vitro and in vivo.

### Cytotoxicity of HFNP@GOX@PFC nanosystem

The cytotoxicity of HFNP@GOX@PFC nanosystem against the HC11 normal breast cells and 4T1 breast cancer cells were both detected by CCK-8 assay. After treatment with a series of concentrations of HFNP@GOX@PFC (from 7.5 to 120 μM of Fe^2+^) for 24 h, HC11 cells showed good cell viability similar to that of the control (Fig. 3f), indicating the good biocompatibility. Only the moderate cytotoxicity was exhibited in the largest drug dosage treatment (240 μM and 480 μM). On the contrary, 4T1 tumor cells showed obvious Fe^2+^ dose-dependent cytotoxicity when the concentration ranges from 30 μM to 480 μM. More importantly, the cell viability dropped sharply upon the Fe^2+^ dose exceed to 60 μM (p < 0.01), and the IC50 was 59.18 × 10^–6^ M. It was suggested that the HFNP@GOX@PFC nanosystem possesses the superior cytotoxicity against tumor cells, and the friendly cell viability for normal cells. The reasons may be attributed to the HA-endowed actively targeting endocytosis and the cascade activation of the CDT combined starvation therapy in vitro, leading to the effective tumor killing.

The time-dependent tumor killing effect of HFNP@GOX@PFC was further investigated using CCK-8 assay as well. Typically, 4T1 cells were co-cultured with different treatment groups for 24, 48 and 72 h, respectively. Compared with control (PBS), free PFC treatment displayed no significant cytotoxicity (Fig. 3g), indicating the biosafety of PFC. Both GOX and HFNP caused the moderate cytotoxicity after treatment for 24 h, and the tendency became more obvious as the incubation time extended to 48 h, owing to the inherent starvation therapy mediated by GOX and CDT cytotoxicity executed by Fe^2+^-mediated Fenton reaction [10, 22]. Moreover, GOX loaded groups (HFNP@GOX and HFNP@GOX@PFC) showed higher cytotoxicity than free GOX and blank HFNP regardless of incubation time, benefiting from the superior drug delivery efficiency and improved biocompatibility. More importantly, HFNP@GOX@PFC induced the most serious cytotoxicity among all the treatment groups despite of incubation time, and above tendency became more obvious as the incubation time extended to 48 and 72 h, indicating the excellent tumor killing effect and time-dependent cytotoxicity. The reason could be explained that HFNP@GOX@PFC nanosystem with high delivery efficiency could effectively accumulate into tumor cells, and the release of O_2_ supplier PFC, H_2_O_2_ supplier plus starvation inducer GOX and CDT trigger agent Fe^2+^ could collectively kill tumor cells via initiating the cascade amplification of tumor starvation and CDT therapy, leading to the highest tumor damage.

### Tumor-targeted cell uptake of HFNP@GOX@PFC nanosystem

In order to reveal the active targeting endocytosis of HFNP@GOX@PFC nanosystem with high efficiency above mentioned, GOX conjugated with FITC were first loaded into the nanosystem. Subsequently, 4T1 cells were treated with FNP@GOX@PFC and HFNP@GOX@PFC for 12 h, and the cellular uptake degree of nanosystem was distinctly observed by CLSM. As shown in Fig. 3h, both the FNP@GOX@PFC and HFNP@GOX@PFC nanosystem labelled with green fluorescence were uptake by tumor cells, shown by the obvious green fluorescence distributed in the cytoplasm. Furthermore, the amount of HFNP@GOX@PFC endocytosed by tumor cells was significantly higher than that of FNP@GOX@PFC without HA conjugation, which was confirmed by the correspondingly quantitative analysis (about 2.3-fold, p < 0.01, Fig. 3i). The result suggested that HA-modified nanosystem could effectively be endocytosed by 4T1 cells overexpressed HA receptors via the active targeting endocytosis pathway based on the HA receptor-ligand interaction effect, which was consistent with a previous study [16].

### The ROS self-generation and hypoxia suppression of HFNP@GOX@PFC nanosystem

**Fig. 4:**
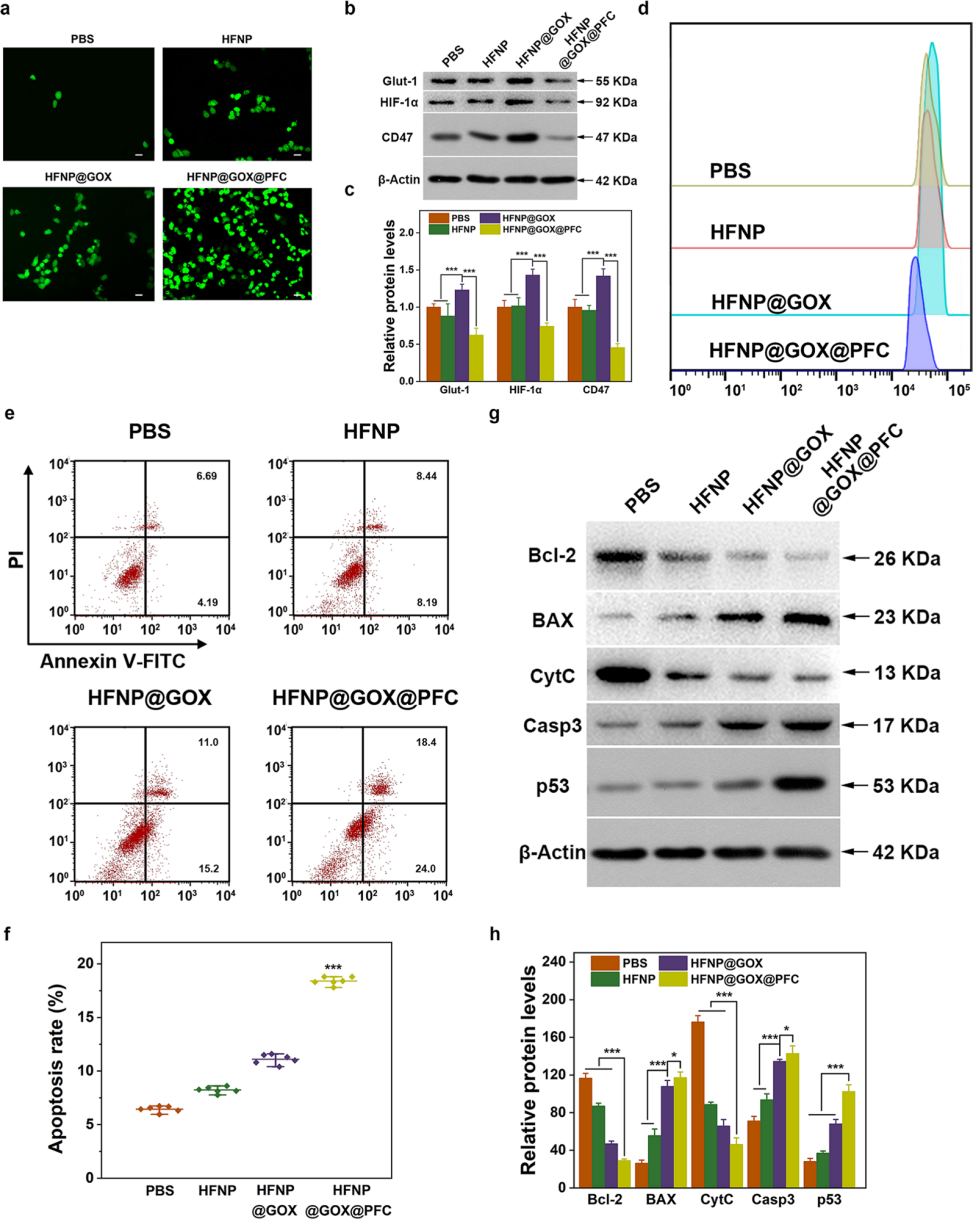
**a** Intracellular ROS images of 4T1 cells after treated with PBS, HFNP, HFNP@GOX & HFNP@GOX@PFC for 24 h. **b** The expression of Glut-1, HIF-1α and CD47 in 4T1 cells examined by Western blotting, **c** corresponding quantitative analysis and **d** CD47 FCM after treatment with PBS, HFNP, HFNP@GOX & HFNP@GOX@PFC for 24 h. **e** Apoptosis and **f** corresponding quantitate analysis of 4T1 cells after above treatments, detected by FCM. **g** Bcl-2, BAX, CytC, Casp3 and p53 relative protein levels and **h** corresponding quantitative analysis of 4T1 cells treated as above, detected by Western blotting. Scale bar: 200 μm. Error bars present as mean ± SD (n = 6)

As the realization of the desired antitumor effect is closely with the activity of GOX and PFC cargoes, which was used to start the designed starvation and Fenton-based CDT therapy in HFNP@GOX@PFC nanosystem. The intratumoral activity of GOX and PFC was subsequently investigated. Since the GOX-mediated tumor starvation and PFC-mediated O_2_ supply and CDT amplification were accompanied by ROS generation and hypoxia suppression, which were respectively investigated in the following study. For one thing, 4T1 cells were treated with administrations and the ROS generation level was monitored by FCM using the typical ROS probe of DCFH-DA. As shown in Fig. 4a, compared with control (PBS), HFNP treatment induced the obvious fluorescence signal of DCF, indicating the generation of ROS, which was owed to the production of ·OH via Fenton reaction mediated by Fe^2+^ [22]. Furthermore, the amount of ROS generation generated by HFNP@GOX was significantly higher than HFNP group, benefitting from the introduction of GOX. Moreover, HFNP@GOX@PFC induced the highest ROS level in tumor cells among all treatments (Additional file 1: Fig. S12), implying the effective ROS self-generation capacity. The reason could be explained that the introduction of O_2_ supplier PFC in HFNP@GOX@PFC nanosystem could provide the endogenous O_2,_ which was the key fuel for GOX-mediated starvation, leading to the dramatically H_2_O_2_ self-generation and ·OH accumulation via the cascade amplification of Fe^2+^-based Fenton reaction. These results directly illustrated that HFNP@GOX@PFC nanosystem is capable of the ROS self-generation features, which was helpful for the improved tumor killing.

For another thing, the hypoxia suppression induced by HFNP@GOX@PFC nanosystem was simultaneously investigated. Since the hypoxia level of tumor cells is positively associated with the expression of the typical hypoxia HIF-1α, CD47 and Glut-1 proteins [8, 23], its expression was thus monitored for evaluation the hypoxia suppression in tumor cells using Western blot and Image J software. After treatment of the above groups for 24 h, there was no obvious difference in the expression of HIF-1α, Glut-1 and CD47 proteins of 4T1 cells between control (PBS) and HFNP group (Fig. 4b), suggesting that the introduction of HFNP without O_2_ supplier PFC and O_2_ consumer GOX loading has no effect on the hypoxia suppression, resulting from the inactivation of O_2_ generation. On the contrary, the proteins expression was significantly up-regulated in H_2_O_2_ supplier of GOX loaded HFNP@GOX group owing to the additional O_2_ exhaustion, which was the indispensable fuel of the catalytic reaction mediated by GOX. Furthermore, HFNP@GOX@PFC group induced the sharply downregulation of these proteins’ expression (p < 0.01, Fig. 4c), compared with other groups. In addition, the FCM result confirmed the above phenomenon, with a significant reduction in CD47 expression on the HFNP@GOX@PFC group (Fig. 4d and Additional file 1: Fig. S13). Benefiting from the relatively higher loading content of PFC than GOX (32.37% *vs.* 2.42%, Additional file 1: Fig. S7 and Fig. S5), the self-supply amount of the endogenous O_2_ generated by PFC in HFNP@GOX@PFC nanosystem is correspondingly higher than that of O_2_ exhaustion mediated by GOX, leading to the effective hypoxia suppression, as shown by the lowest of HIF-1α, Glut-1 and CD47 expression in 4T1 cells. The result further confirmed that the HFNP@GOX@PFC nanosystem not only self-supply O_2_ in tumor cells and relieves its hypoxic state, but also self-generates ROS via initiating the starvation mediated by GOX, and cascade amplify CDT effect mediated by Fe^2+^-based Fenton reaction, leading to the desired tumor killing.

### The in vitro tumor apoptosis of HFNP@GOX@PFC nanosystem

In order to further explore the antitumor effect in vitro, the apoptosis ratio of 4T1 cells treated with above experiment group was subsequently detected by FCM using Annexin V-FITC/PI kit (NeoBioscience, China). As shown in Fig. 4e, blank HFNP group caused a moderate apoptosis ratio compared to control (PBS), attributed to the limited CDT efficiency in presence of the insufficient H_2_O_2_. Furthermore, HFNP@GOX treatment group caused more severe apoptosis compared to HFNP group (p < 0.01, Fig. 4f), owing to the introduction of starvation therapy mediated by GOX. Moreover, the HFNP@GOX@PFC nanosystem induced the highest apoptosis rate compared with other groups, as confirmed by the quantitative analysis (Fig. 4f). The reasons could be explained that the introduction of O_2_ supplier-PFC could effectively self-supply the endogenic O_2_ and relieve intracellular hypoxia, which was conducive to promote the GOX-based starvation reaction and generate the abundant H_2_O_2_, followed by accelerating Mof disassembly, drug release and cascade boosting CDT, consequently leading to the effective tumor apoptosis. Subsequently, the expression levels of the typical proteins closely associated with apoptosis pathway were measured with western blotting and qPCR assays for revealing the apoptosis mechanism induced by HFNP@GOX@PFC nanosystem [24, 25]. Compared to control, HFNP, HFNP@GOX and HFNP@GOX@PFC groups certainly downregulated the expression of anti-apoptotic BCL-2 & CytC genes and upregulated the expression of pro-apoptotic BAX, Casp3 genes & p53 proteins (Fig. 4g, h and Additional file 1: Fig. S14), in an order of HFNP < HFNP@GOX < HFNP@GOX@PFC, confirming again the superior tumor killing efficiency of HFNP@GOX@PFC nanosystem. It was explained that taking the combined advantages of the self-supply O_2_ via PFC, the dramatic H_2_O_2_ self-generation via GOX-based catalytic reaction, the ·OH accumulation via the cascade amplification of Fenton reaction, and the improved delivery efficiency via HA-targeted strategy, HFNP@GOX@PFC nanosystem induced the most serious cell apoptosis in vitro.

### Re-education of macrophage and anti-metastasis in vitro

**Fig. 5:**
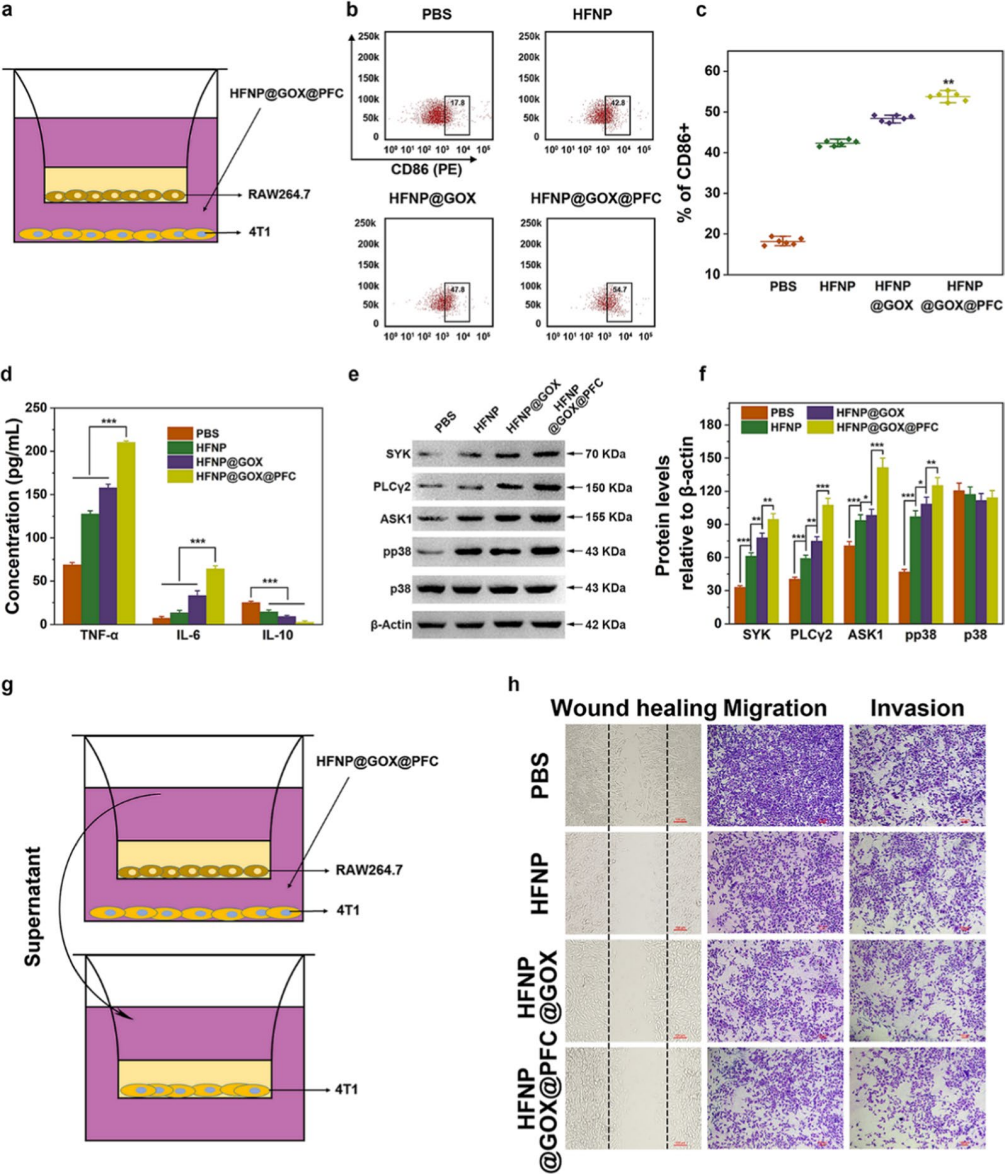
**a** Schematic diagram of tumor cells and macrophages co-culture assay via Transwell. **b** The percentage of CD86 positive cells and **c** corresponding quantitate analysis. 4T1 cells seeded on the lower station were cultured with above treatment groups for 24 h. RAW264.7 cells were co-stained with PerCP-Cy5.5-CD11b, AF488-F4/80 and PE-CD86, followed by FCM analysis. **d** TNF-α, IL-6 and IL-10 cytokines concentration of supernatant in 4T1 and RAW 264.7 cells co-culture system, detected by ELISA. **e** The expression of SYK, PLCγ2, ASK1, pp38 and p38 in RAW264.7 cells examined by Western blotting and **f** corresponding quantitative analysis using Image J software. **g** Schematic diagram and results **h** of wound healing, migration and invasion assays of 4T1 cells via co-culture system. Scale bar: 100 μm. Error bars present as mean ± SD (n = 6)

As the level of H_2_O_2_ is closely associated with the polarization of tumor-associated macrophages (TAMs) [8], and HFNP@GOX@PFC nanosystem actually possesses the ROS self-generation features, as revealed by above result (Fig. 3c–e and Fig. 4a). Thus, the HFNP@GOX@PFC nanosystem may play a key role in the re-education of TAMs. Based on above considerations, 4T1 tumor cells and IL4-pretreated RAW264.7 macrophages were firstly co-cultured in the Transwell, and 4T1 cells were then treated with various administrations, FCM was finally used to monitor the re-education degree of macrophages. The co-culturation platform was illustrated in Fig. 5a, and the gate strategy of FCM was shown in Additional file 1: Fig. S15. After above administrations, the percentage of M1 macrophages were all significantly increased compared with control (PBS), in an order of HFNP < HFNP@GOX < HFNP@GOX@PFC (p < 0.01, Fig. 5b, c), indicating the effective TAMs polarization. It was attributed to the difference in ROS generation capacity (Fig. 3c–e and Fig. 4a). Notably, the introduction of O_2_ supplier PFC and H_2_O_2_ supplier GOX in HFNP@GOX@PFC nanosystem could provide the sufficient fuels (O_2_ and H_2_O_2_) for GOX-mediated catalytic reaction and Fe^2+^-based Fenton reaction, leading to the abundant production of ROS (including H_2_O_2_ and ·OH) and the highest re-education number of TAMs.

As the polarization of TAMs is positively associated with the special cytokine secretion [26]. In general, TNF-α and IL-6 are considered to be the special markers of M1 macrophages, and IL-10 is considered to be the special marker of M2 macrophages [27]. In order to further clarify the re-education degree of TAMs, Elisa assay was employed to investigate expression change of above cytokine mentioned. As shown in Fig. 5d, the expression of TNF-α and IL-6 in HFNP and HFNP@GOX groups were obviously higher than control (PBS), and HFNP@GOX@PFC group induced the highest expression among all the treatments. On the contrary, the expression of IL-10 in treatment groups displayed the decreased tendency, in an order of HFNP@GOX@PFC < HFNP@GOX < HFNP, and HFNP@GOX@PFC group caused the lowest expression than other treatment groups, confirming again the great TAMs polarization capability. It was consistent with previous FCM analysis on the re-education of TAMs (Fig. 5b). These results directly confirmed that HFNP@GOX@PFC nanosystem is capable of the re-education of TAMs features, which was helpful for the immune activation and tumor killing.

As mentioned above, we have confirmed that HFNP@GOX@PFC nanosystem can induce the re-education of TAMs, but the molecular mechanism needs further exploration in the following study. It is reported that ROS may serve as secondary messenger to promote the regulation of downstream pathways, such as NF-κB and MAPK, which are closely associated with macrophage polarization [28]. Therefore, 4T1 and RAW264.7 cells were co-cultured in the Transwell chamber and treated with HFNP, HFNP@GOX and HFNP@GOX@PFC, respectively. The expression of SYK, PLCγ2, ASK1, pp38 and p38 key proteins associated with NF-κB and MAPK pathways were measured with Western blot. As shown in Fig. 5e, the expression of SYK, PLCγ2, ASK1 and pp38 positive regulatory proteins associated with TAMs polarization in HFNP group were obviously higher than control (PBS), and HFNP@GOX group displayed higher expression than HFNP group. Importantly, HFNP@GOX@PFC group induced the highest expression among all the treatments (p < 0.01, Fig. 5f). It was attributed to the highest ROS generation of HFNP@GOX@PFC nanosystem. These results proved that the molecular mechanism of TAMs re-education of HFNP@GOX@PFC nanosystem was indeed achieved by ROS-activated NF-κB and MAPK signaling pathways.

Since TAMs could involve directly or indirectly in the metastasis of malignant tumor [29], we additionally investigated the wound healing, migration and invasion behaviors of 4T1 cells in Transwell co-culturation system containing RAW 264.7 cells after above administrations (Fig. 5g), detected by the optical microscope. As for the wound healing assay, compared with the control group (PBS), the wound closure rate of HFNP, HFNP@GOX and HFNP@GOX@PFC groups obviously decreased to 85.9%, 68.9% and 42.6% (Fig. 5h and Additional file 1: Fig. S16), respectively. It was due to that the addition of PFC accelerated the generation of ROS and improved the re-education of macrophages, and the pro-inflammatory M1 macrophages can suppress the growth and metastasis of tumor cells and anti-inflammatory M2 macrophages can be conducive to tumor metastasis and progress [30]. As for migration and invasion assay, compared with control, HFNP, HFNP@GOX and HFNP@GOX@PFC groups could dramatically down-regulate migration ratio to 73.3%, 63.4% and 55.2% (Fig. 5h and Additional file 1: Fig. S17), and invasion rate to 94.1%, 79.9% and 73.9% (Fig. 5h and Additional file 1: Fig. S18) as well, suggesting again the effective anti-tumor invasion and migration capability of HFNP@GOX@PFC nanosystem. The above results collectively suggested that the HFNP@GOX@PFC uptake by tumor cells with high efficiency can significantly self-supply H_2_O_2_ and O_2_ via delivering GOX and PFC, which could not only accelerate the disintegration of the HFNP@GOX@PFC nanosystem and drug release, but also cascade amplify the GOX-mediated tumor starvation and CDT therapy via Fe^2+^-based Fenton reaction with re-educated TAMs, consequently devoting to the effective antitumor effect in vitro.

### In vivo antitumor effect of HFNP@GOX@PFC nanosystem

**Fig. 6:**
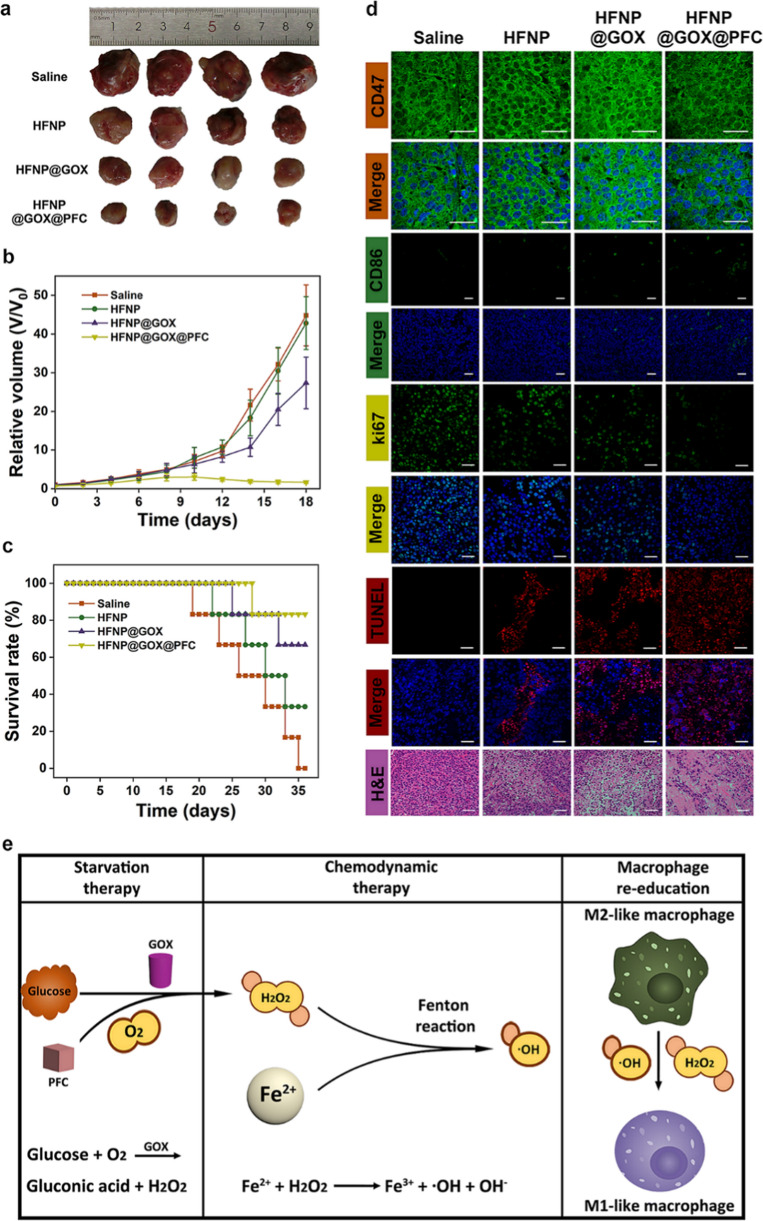
**a** Representative optical photos of tumors after treatment with saline, HFNP, HFNP@GOX and HFNP@GOX@PFC for 18 days, respectively. **b** Relative tumor volume and **c** survival rate after above treatments. **d** IFC images of CD47 and CD86, H&E, TUNEL and ki67 staining images for tumor tissue. **e** Schematic illustration of HFNP@GOX@PFC nanosystem for combined CDT and starvation therapy with TAMs re-education. Scale bar: 100 μm. Error bars present as mean ± SD (n = 6)

Inspired by the excellent antitumor effect of the HFNP@GOX@PFC nanosystem in vitro, we further studied its antitumor effect in vivo in 4T1 tumor-bearing mouse models. After various administrations, the tumor volume, body weight and survival rate of the tumor-bearing mouse were significantly changed. In detail, HFNP group induced moderate tumor growth inhibition (Fig. 6a) compared with control (saline), attributing to the insufficient CDT. Furthermore, the inhibition effect of tumor growth in HFNP@GOX group was higher than that of HFNP, owing to the additional starvation therapy mediated by GOX. Moreover, the HFNP@GOX@PFC group generated the most serious tumor growth inhibition, resulting from the combined effect of GOX-provided starvation therapy and cascade amplification of CDT mediated by Fe^2+^-based Fenton reaction. Additionally, the re-education of TAMs mediated by HFNP@GOX@PFC nanosystem may also play an essential role in the overall antitumor effect, which was investigated in the following section. The tumor volume analysis (Fig. 6b) further confirmed the similar inhibition tendency of the tumor growth. More importantly, compared with other groups, HFNP@GOX@PFC group significantly prolonged the survival time of tumor-bearing mice (Fig. 6c) without weight loss (Additional file 1: Fig. S19), displaying the highest survival rate as 83.3% upon 36 days, suggesting the excellent anti-tumor efficacy and good biosafety in vivo.

Subsequently, H&E, TUNEL and immunofluorescence staining (IFC) assays were performed to further reveal the tumor killing in vivo. Compared with saline, HFNP, HFNP@GOX and HFNP@GOX@PFC groups all led to a different degrees of tumor tissues damage, in an order of HFNP < HFNP@GOX < HFNP@GOX@PFC, demonstrated by the chromatin condensation and distinctly tissue structure damaged in H&E images, and abundant magenta dots labelled the damaged DNA in TUNEL images (Fig. 6d). Besides, the IFC analysis of Ki67 in the tumor tissues also verified that HFNP@GOX@PFC group could effectively reduce the expression of Ki67 (Fig. 6d), confirming again the excellent antitumor effect in vivo of HFNP@GOX@PFC nanosystem. It may be attributed to the combined therapy advantages of the GOX-mediated starvation therapy and cascade amplification of CDT provided by Fe^2+^-based Fenton reaction with potential TAMs re-education. The results collectively suggested again that HFNP@GOX@PFC nanosystem certainly was capable of the excellent antitumor efficiency in vivo.

In order to clarify the antitumor mechanism of HFNP@GOX@PFC nanosystem, we first investigated hypoxia suppression in vivo, as the level of endogenous O_2_ was close with GOX-mediated starvation effect, and the H_2_O_2_ produced by GOX also affected the subsequent CDT and the overall antitumor effect. The IFC analysis of hypoxia marker CD47 in tumor sections after various administrations was investigated for characterizing the hypoxia suppression in vivo. As shown in Fig. 6d, the expression of CD47 in HFNP group exhibited the relatively high dose, as shown by the bright green fluorescence, and it was no different from that of control (saline) group, indicating the inherent tumor hypoxia and the little hypoxia suppression without additional O_2_ supply or exhaustion, Furthermore, O_2_ consumer and H_2_O_2_ supplier of GOX loaded HFNP@GOX group displayed the much higher expression of CD47 than HFNP group, and the reason could be attributed the fact that the GOX indeed induces the H_2_O_2_ generation with O_2_ deletion via the catalytic reaction, leading to the increased hypoxia in the tumor, as shown by the higher expression of CD47. Importantly, HFNP@GOX@PFC group with high PFC loading content (32.37%) displayed the lowest expression of CD47 among all groups (p < 0.01, Additional file 1: Fig. S20), implying the effective hypoxia suppression. It was explained that the abundant introduction of O_2_ supplier-PFC could significantly relieve the hypoxia of tumor cells. These results were consistent with the western blotting and FCM analysis of CD47 expression in vitro (Fig. 4b–d and Additional file 1: Fig. S13). Therefore, the results collectively suggested that the HFNP@GOX@PFC nanosystem could self-supply O_2_ and H_2_O_2_ with high efficiency, which could not only relieve tumor hypoxia, but also contribute to the realization of the cascade amplification of CDT and GOX-mediated tumor starvation, leading to the most effective antitumor effect in vivo.

We next studied the polarization degree of TAMs in vivo to further reveal the antitumor mechanism of HFNP@GOX@PFC nanosystem, as the self-generation of ROS could actually influence the re-education of TAMs and devote to the overall antitumor performances in vivo. Accordingly, the IFC analysis of the typical marker CD86 labelled M1 macrophages in tumor sections after various administrations was detected by CLSM for evaluating the polarization degree of TAMs in vivo. As shown in Fig. 6d, compared with control (saline), HFNP induced the moderate TAMs re-education, as shown by the suitable up-regulation of the CD86 expression, owing to the limited CDT and insufficient ROS generation. Moreover, the number of M1-macrophages in HFNP@GOX group was obviously higher than HFNP group, attributing to the additional H_2_O_2_ generation mediated by GOX and its promotion effect on TAMs polarization. More importantly, HFNP@GOX@PFC group induced the highest TAMs polarization, shown by the brightest fluorescence of CD86 among all treatment groups (Additional file 1: Fig. S21). It was explained that the delivery of PFC could significantly promote the GOX-mediated tumor starvation with the abundant H_2_O_2_ generation via supplying plenty of O_2_, which could not only accelerate MOF disassembly, drug release, and cascade amplify CDT via providing the sufficient fuel of Fe^2+^ and H_2_O_2_, but also effectively enhance the polarization of TAMs, consequently contributing to the desired antitumor effect in vivo.

Finally, the blood biosafety of the tumor-bearing mouse was investigated for evaluating the overall biosafety of the HFNP@GOX@PFC nanosystem in vivo by hematology analysis. As shown in Additional file 1: Fig. S22, compared with control (saline), most of the measured parameters for HFNP@GOX@PFC treated displayed no discernible changes. Excitingly, the major tissues (heart, liver, spleen, kidney and lung) also had no obvious damage in HFNP@GOX@PFC group (Additional file 1: Fig. S23), and the body weight steadily increased with the feeding time as well (Additional file 1: Fig. S19), confirming the good biocompatibility of the HFNP@GOX@PFC nanosystem. Based on the above results, the excellent antitumor effect of the HFNP@GOX@PFC nanosystem with good biosafety could be explained as follows (Fig. 6e): (1) the HA-modified functional nanosystem HFNP@GOX@PFC based on ROS- responsive degradable Fe-MOF could effectively uptake by 4T1 cells via the HA targeting endocytosis pathway; (2) the endocytosed nanosystem could rapidly degradable and release cargoes of the O_2_ supplier PFC, H_2_O_2_ supplier plus starvation inducer GOX and CDT trigger agent Fe^2+^ in response to the relatively high concentrations of ROS in the cytoplasm; (3) GOX from HFNP@GOX@PFC nanosystem could effectively consume intracellular glucose and O_2_ to starve tumor cells and generate plenty of H_2_O_2_, and the PFC from nanosystem could self-supply abundant O_2_ for further promoting GOX-mediated tumor starvation and H_2_O_2_ generation. More importantly, the self-generation of H_2_O_2_ could not only accelerate HFNP@GOX@PFC disassembly and cargoes release, but also cascade amplify CDT effect via Fe^2+^-based Fenton reaction; (4) ROS generated by above cycle reactions could excitingly re-educate TAMs, which could further improve tumor killing. The above works could collectively devote to the superior tumor therapy effect. The in vitro and in vivo data suggested that HFNP@GOX@PFC nanosystem could achieve effective antitumor therapy with good biocompatibility by the cascade amplification of tumor starvation mediated by GOX and CDT with TAMs re-education.

## Conclusions

In summary, a tumor-targeted HFNP@GOX@PFC nanosystem composed of ROS-cleaved Fe-based metal–organic framework, HA, GOX and PFC have been developed for tumor cascade amplified starvation and CDT with TAMs re-education. The nanosystem possesses several advantages: (1) HFNP@GOX@PFC nanosystem could specially accumulate in the tumor site via the HA receptor-ligand mediated targeting endocytosis pathway; (2) HFNP@GOX@PFC nanosystem uptake by tumor cells is then disassembled in response to ROS in the cytoplasm of tumor cells, leading to the release of O_2_-supplier PFC, H_2_O_2_ supplier and starvation inducer GOX, and CDT trigger agent Fe^2+^; (3) PFC can reduce the hypoxia and promote the initiation of catalytic reaction mediated by GOX. GOX can competitively react with intracellular glucose and sufficient O_2_ to starve tumor and produce abundant H_2_O_2_, which could further facilitate nanosystem disassembly, drug release and oxidative tumor killing. Fe^2+^ reacts with H_2_O_2_ to cascade amplify CDT and induce effective tumor damage; (4) ROS generated by the above cycle could additionally boost tumor immune response via re-educating TAMs by activating NF-κB and MAPK signal pathways, leading to the superior tumor therapy effect. The in vitro and in vivo studies had shown that HFNP@GOX@PFC nanosystem had excellent stability, biocompatibility and antitumor effect by combining starvation and CDT with TAMs re-education. Given the above advantages, this work exhibits that HFNP@GOX@PFC nanosystem is a potent self-motivation vehicle combining the cascade amplification of starvation and CDT with TAMs re-education against malignant tumors.

## Materials and methods

### Materials

Ferrous acetate (FeAc_2_·4H_2_O) and 2,2'-[propane-2,2-diylbis(thio)]diacetic acid were respectively purchased from Sigma-Aldrich (Beijing, China) and Ruixi Bio-Technology Co., Ltd (Xi’an, China). Hyaluronic acid (HA, 36 kDa) was offered by Shandong Freda Biochem Co., Ltd (Shandong, China). Perfluorohexane (PFC), N-hydroxy succinimide (NHS), 1-(3-dimethylaminopropyl)-3ethylcarbodiimide hydrochloride (EDC) and Glucose oxidase (GOX) were purchased from Macklin Biochem-Technology (Xi’an, China) and Yuanye Bio-Technology Co., Ltd (Shanghai, China). Interleukin-6 (IL-6), Interleukin-10 (IL-10) and tumor necrosis factor-α (TNF-α) ELISA kits were purchased from Elabscience Biotechnology Co., Ltd (Wuhan, China). Anti-CD11b, anti-F4/80 and anti-CD86 were purchased from BioLegend Co., Ltd (California, America). All the reagents were analytical grade.

### Preparation of HFNP@GOX@PFC nanosystem

Firstly, FeAc_2_·4H_2_O (1 mmol) and 2,2'-[propane-2,2-diylbis(thio)]diacetic acid (1 mmol) were dissolved in 15 mL N, N-Dimethylformamide (DMF) in presence of acetic acid (1 mL), the mixture solution was then placed into a Teflon-liner steel autoclave at 65 °C for 2 h. Next, the obtained sediment was collected by centrifugation at 5000 rpm min^−1^ for 10 min. The sediment was added to 100 mL ultrapure water overnight, and the blank Fe-MOF (named FNP) was harvested via centrifugation.

Subsequently, FNP (20 mg), EDC (115 mg) and NHS (69 mg) were dissolved in 10 mL of PBS (pH 6.0). After stirring for 1.5 h, ethylene diamine (1.67 mL) was added into above mixture at room temperature and continued for 24 h. The functionalized FNP was then collected and mixed with GOX (1.5 mg) and PFC (500 μL) in PBS (10 mL). Above mixture was stirred overnight at room temperature. For one thing, above solution was centrifuged and obtained the drug loaded FNP without HA modification (donated FNP@GOX@PFC). For another thing, 60 mg of HA, 230 mg of EDC and 138 mg of NHS were dissolved in 10 mL of PBS and stirred for 1.5 h. Next, 30 mg of FNP@GOX@PFC was added into above solution and stirred for another 24 h. The final product was obtained by centrifugation and dried by lyophilization, donated as HFNP@GOX@PFC. Notably, the blank nanosystem without drug loading was donated as HFNP.

Meanwhile, the drug loading content (DLC) and efficiency (DLE) of GOX in HFNP@GOX@PFC nanosystem were monitored by the ultraviolet and visible spectrophotometry (UV–vis) at 276 nm and the corresponding standard curve, according to previous literature [8]. Besides, the DLC and DLE of PFC in HFNP@GOX@PFC were determined by gas chromatography (GC). Briefly, 50 μL of HFNP@GOX@PFC was dissolved in 50 μL of acetonitrile and immersed in ultrasonic for 10 s. Then, 1,1,1,3,3-pentafluorobutane (200 μL) was added to the solution under ultrasonic for 10 s. Afterward, the mixture was centrifuged at 1500 g for 2 min at 4 °C and stored at -20 °C overnight. 0.5 μL of sample solution was then detected by the GC and calculated the concentration of PFC based on its standard curve. Notably, pure PFC was treated in the same way to obtain the standard curve. DLC and DLE were calculated as follows:

$${\text{DLC }} = \, \left( {{\text{WT }}{-}{\text{ WF}}} \right) \, /{\text{ WNP }} \times { 1}00\%$$

$${\text{DLE }} = \, \left( {{\text{WT }} - {\text{ WF}}} \right) \, /{\text{ WT }} \times { 1}00\%$$

where WT is the total weight of GOX or PFC fed, WF is the weight of nonencapsulated free GOX or PFC, and WNP is the weight of drug loaded nanosystem.

### Characterization of materials

The size and zeta potential of nanoparticles are measured by dynamic light scattering (DLS). The morphology and structure of FNP and HFNP nanosystem were characterized by transmission electron microscopy (TEM) and X-ray diffraction (XRD), respectively. The conjugation and functionalization processes of HFNP nanosystem were analyzed by Fourier Transform Infrared Spectrometer (FTIR). The drug release behavior of HFNP@GOX@PFC nanosystem was characterized by UV–vis spectrophotometry.

### Stability of HFNP@GOX@PFC

Typically, HFNP@GOX@PFC nanosystem (1 mg mL^−1^) was firstly co-cultured with 10% FBS in PBS solution at 37 °C for various time intervals (0 h, 2 h, 4 h, 8 h, 10 h, 12 h and 24 h), DLS was used to detect its size and potential change for evaluation the stability of HFNP@GOX@PFC in the short term. As for long-term stability, HFNP@GOX@PFC nanosystem (1 mg mL^−1^) were cultured in PBS solution at 4 °C for various time intervals (1 d 3 d, 5 d, 7 d, 9 d, 11 d and 14 d), DLS was used to monitor the stability of nanosystem using above same method.

### Drug release behavior

HFNP@GOX@PFC nanoparticles (1 mg) were dispersed into 5 mL PBS solution containing different concentrations of H_2_O_2_ (0 mM, 0.1 mM and 1 mM) and transferred to dialysis bag (MWCO = 14 kDa). Then, the dialysis bag was immersed in 29 mL PBS solution under gently stirring, 0.8 mL of PBS was taken out at the certain time points (0.5 h, 1 h, 2 h, 4 h, 8 h, 12 h, 24 h and 48 h) and supplemented with the same amount of fresh PBS containing different doses of H_2_O_2_. The release content of GOX and Fe^2+^ were respectively monitored by the analyzed UV–vis at 276 nm and 506 nm using o-phenanthroline method [16].

### Self-supplying H_2_O_2_ detection and self-amplified generation of ·OH

In order to detect the self-generation of H_2_O_2_ from HFNP@GOX@PFC nanosystem, PBS, HFNP, HFNP@GOX and HFNP@GOX@PFC were respectively added into 8 mL PBS containing glucose (1 mg mL^−1^). The mixture was then incubated at room temperature under gentle stirring, 0.8 mL of PBS was taken out at 2 h and detected by UV–vis spectrophotometry for characterizing the generation of H_2_O_2_ at 240 nm. Besides, the samples harvested from HFNP@GOX@PFC treatment at the certain time points (0 h, 1 h, 2 h, 4 h and 6 h) were also monitored by the UV–vis spectrophotometry for investigating the time-dependent H_2_O_2_ generation.

For the self-amplification generation of ·OH detection, PBS (1 mL), HFNP (1 mg), HFNP@GOX (1 mg), HFNP@GOX@PFC (1 mg) and HFNP@GOX@PFC (1 mg) plus glucose (1 mg mL^−1^) were respectively mixed with 0.1 mM H_2_O_2_ solution. The above mixture was then stirred under room temperature for 2 h. Next, the samples were detected by UV–vis spectrophotometry at 550 nm using Hydroxyl Free Radical assay kit (Beorebo), according to the manufacturer's protocols.

### Cell culture

4T1 breast cancer cells, HC11 mammary gland cells and RAW264.7 macrophages are supplied by Cell Bank of Chinese Academy of Sciences (Shanghai, China). Cells were cultured in Dulbecco's modified Eagle’s medium (DMEM) supplemented with 10% (v/v) fetal bovine serum (FBS, Gibco) and 1% (w/v) penicillin (100 U mL^−1^)/streptomycin (100 μg mL^−1^) with 5% CO_2_ at 37 °C.

### In vitro cytocompatibility and cytotoxicity

As for the dose-dependent cytotoxicity, 4T1 cells and normal HC11 cells seeded on the 24-well plates (1 × 10^4^ cells well^−1^) were respectively treated with HFNP@GOX@PFC at different doses (equivalent of 0 μM, 7.5 μM, 15 μM, 30 μM, 60 μM, 120 μM, 240 μM, 480 μM Fe^2+^) for 24 h. The medium was replaced by the mixture of fresh medium (200 μL) and CCK-8 (20 μL) and incubated for another 2 h. Finally, the supernatant was translated to 96-well plates and the absorbance was recorded at 450 nm by spectrophotometric microplate reader (Bio-Rad 680, USA). IC50 was calculated according to the cytotoxicity result.

As for the time-dependent cytotoxicity, 4T1 cells were treated with PBS, GOX (1.5 μM), PFC (4.85 μM), HFNP (3.3 mg mL^−1^), HFNP@GOX (3.4 mg mL^−1^, equivalent of 59.18 μM Fe^2+^) and HFNP@GOX@PFC (5.06 mg mL^−1^, equivalent of 59.18 μM Fe^2+^) for 24 h, 48 h and 72 h, respectively. CCK-8 kit was used to determine the corresponding cell viability, according to the above method.

### Tumor targeting cellular uptake in vitro

In order to visualize the tumor targeting endocytosis of 4T1 cells against nanosystem, 4T1 cells (1 × 10^5^ cells well^−1^) inoculated in 6-well plates were co-cultured with FNP@GOX@PFC and HFNP@GOX@PFC for 12 h, respectively. Notably, GOX loaded into the above nanosystem was pre-labeled with FITC before drug loading, thus exhibiting green fluorescence upon laser irradiation. Then, the cell was washed, fixed and permeabilized, the nuclei were stained with DAPI and the cytoskeletons were stained with Tetramethyl Rhodamine Isothiocyanate (TRITC). Finally, these cells were imaged by confocal laser scanning microscopy (CLSM) to observe the tumor targeting endocytosis behavior and the cellular distribution of the nanosystem.

### Cellular ROS detection

Briefly, 4T1 cells (1 × 10^5^ cells well^−1^) were inoculated in 6-well plates and treated with PBS, HFNP (3.3 mg mL^−1^), HFNP@GOX (3.4 mg mL^−1^) and HFNP@GOX@PFC (5.06 mg mL^−1^) for 24 h. DCFH-DA probes were used to mark the generation of intracellular ROS, which was imaged by CLSM and quantitively analyzed by Image J software. The detailed procedures were referred to previous literature [31].

### The Flow cytometry (FCM) analysis of the hypoxia, TAMs polarization and apoptosis

For the hypoxia suppression study, 4T1 cells (1 × 10^5^ cells well^−1^) inoculated in 6-well plates were treated with PBS, HFNP (3.3 mg mL^−1^), HFNP@GOX (3.4 mg mL^−1^) and HFNP@GOX@PFC (5.06 mg mL^−1^) for 24 h. The cells were then washed with PBS, collected by centrifugation, followed by staining with CD47 (hypoxia mark) antibody labelled with PE. Finally, the cells were monitored by FCM.

For TAMs polarization study, RAW264.7 was pre-treated with IL-4 for 24 h, and then co-cultured with 4T1 cells in the Transwell chamber. Meanwhile, the 4T1 cells seeded in the lower chamber were treated with PBS, HFNP, HFNP@GOX and HFNP@GOX@PFC for 24 h, respectively. Subsequently, RAW264.7 cells seeded in the upper chamber were collected, co-stained with PerCP-Cy5.5-CD11b, AF488-F4/80 and PE-CD86 antibodies, followed by FCM analysis.

As for the apoptosis assay, 4T1 cells (1 × 10^5^ cells well^−1^) inoculated in 6-well plates were treated with PBS, HFNP, HFNP@GOX and HFNP@GOX@PFC for 24 h, respectively. The cells were then washed with PBS twice, collected by centrifugation, followed by staining with Annexin V-FITC/PI kit, the detailed procedures could refer to our previous literature [32].

### Enzyme linked immunosorbent assay (ELISA)

Briefly, the RAW264.7 cells were seeded in the upper chamber of the Transwell, and the 4T1 cells seeded in the lower chamber were treated with PBS, HFNP, HFNP@GOX and HFNP@GOX@PFC for 24 h, respectively. The supernatant was collected and measured the level of TNF-α, IL-6 and IL-10 to evaluate the polarization degree of TAMs using ELISA Kit (Elabscience), according to the manufacturer's protocols.

### Western blot analysis

4T1 cells (1 × 10^5^ cells well^−1^) inoculated in 6-well plates were incubated with PBS, HFNP, HFNP@GOX and HFNP@GOX@PFC for 24 h, respectively. Subsequently, cells were lysed, collected and determined the total protein concentration using BCA kit. The expression of p53 protein was measured using Western blot and Image J software. Besides, 4T1 cells treated with PBS, HFNP, HFNP@GOX and HFNP@GOX@PFC for 24 h were also collected and analyzed the expression of HIF-1α, Glut-1 and CD47 protein using above method.

For another thing, RAW264.7 and 4T1 cells were co-cultured in the Transwell chamber, and the 4T1 cells seeded in the lower chamber were treated with PBS, HFNP, HFNP@GOX and HFNP@GOX@PFC for 24 h, respectively. Then RAW264.7 cells were collected to analyze the expression levels of SYK, PLCγ2, ASK1, pp38 and p38 proteins. The detailed experiment procedures were according to previous literature [33].

### Quantitative real-time polymerase chain reaction (qPCR) analysis

4T1 cells (1 × 10^5^ cells well^−1^) inoculated in 6-well plates were incubated with PBS, HFNP, HFNP@GOX and HFNP@GOX@PFC, respectively. After 24 h of incubation, the total RNA was extracted with Trizol reagent, and then reverse transcription using the reverse transcription Kit (TransGen Biotech). The qPCR was carried out for analysis of the mRNA level of BAX, BCL-2, Caspase-3 (Casp3) and Cytochrome C (CytC) according to previous literature [34]. The sequences of primers were listed in Additional file 1: Table S1.

### Wound healing, migration and invasion assays

RAW264.7 and 4T1 cells were first co-cultured in the Transwell chamber, and the 4T1 cells seeded in the lower chamber were treated with PBS, HFNP, HFNP@GOX and HFNP@GOX@PFC for 24 h, respectively. The supernatant of the co-culture system containing various cytokines secreted by TAMs was collected and donated as Solution 1 for further use.

As for the wound healing assay, 4T1 cells (1 × 10^5^ cells well^−1^) were inoculated in 6-well plates. After the cell density reached 70–80%, a p200 pipette tip was used to produce a scratching wound. Next, the cell medium was removed and added above collected Solution 1 for 24 h co-incubation. The wound healing levels were finally imaged by a microscope (Olympus, Japan).

As for migration and invasion assays, 4T1 cells (1 × 10^5^ cells well^−1^) inoculated in 6-well plates were firstly treated with Solution 1 for 24 h. On the one hand, 4T1 cells (5 × 10^4^ cells) harvested from above system were then seeded in the upper chamber of the Transwell and cultured with serum-free medium, 500 μL of the complete medium was added into the lower chambers. On the other hand, 4T1 cells harvested from above system were seeded in the upper chamber of Transwell pre-coated with Matrigel (BD Biosciences), the medium was chosen as the serum-free medium, and 500 μL of the complete medium was added into the lower chambers. After treatment for 48 h, the cells on the upper station of the chamber were wiped, while cells on the lower surface came from above two systems were stained with crystal violet and observed by microscope to evaluate the migration and invasion level of tumor cells. The detailed experiment procedures were referred to a previous study [35].

### Construction of the 4T1-bearing mice and in vivo antitumor efficacy

All animal experiments were strictly conducted in accordance with the Institutional guidelines for animal experimentation and were approved by the Animal Ethics Committee of the Northwestern Polytechnical University. BALB/c mice (5–6 weeks old) were bought from Beijing Institution for Drug Control, China. The model of tumor-bearing mice was constructed by subcutaneous injection of 4T1 cells (1 × 10^6^) into the right flank groin of the Balb/c mice. After the tumor size reached about 50 mm^3^, tumor-bearing mice were randomly divided into 4 groups (n = 6) and intravenously injected with Saline, HFNP, HFNP@GOX and HFNP@GOX@PFC with an equal amount of Fe^2+^ (60 μmol kg^−1^), respectively. The above treatments were repeated 3 times per week. The body weight and tumor volume were recorded every 2 days. The calculation formula of tumor volume was as follows: tumor volume = ab^2^/2 (a: the maximum dimension of the tumor, b: the minimum dimension of the tumor). The mice were euthanized after 18 days, blood was collected for analyzing the biochemical blood index. The survival rate of mice was monitored for another 18 days after the last administration.

### In vivo histological examination and immunofluorescence assay

After 18-days treatment, the tumor and major tissue including heart, liver, spleen, kidney and lung were collected and sliced for H&E examinations. The morphological changes of these organs after administration were observed by microscope. Besides, the tumor sections were also stained with TUNEL apoptosis detection kit (Beyotime) and imaged by CLSM. As for the immunofluorescence study, tumor sections were blocked with 5% bovine serum albumin, incubated with CD47, CD86 or Ki67 antibodies, and then stained with Alexa Fluor 488-labeled IgG according to the manufacturer's instructions (Immunol Fluorescence Staining Kit, Beyotime, China). Sections were then stained with DAPI and imaged by CLSM.

### Statistics analysis

We performed the statistical analysis with the software of Origin (version 9.0) through Student’s t-test and one-way analysis of variance (ANOVA). All data were expressed as means ± standard deviation (SD). P values of * P < 0.05, ** P < 0.01 and *** P < 0.001, respectively. The confidence levels of 95% and 99% were regarded as a significant difference.

## Supplementary Information

**Additional file 1: Fig. S1.** Surface Zeta potential of FNP and HFNP. **Fig. S2.** XRD of FNP and HFNP. **Fig. S3.** H-NMR of FNP and HFNP. **Fig. S4.** FTIR of FNP and HFNP. **Fig. S5.** Linear relationships between the UV-vis absorbance and the concentration of GOX. **Fig. S6.** Efflux time of pure PFC and HFNP@GOX@PFC nanoparticles. **Fig. S7.** Linear relationships between the GC intensity and the concentration of PFC. **Fig. S8.** TEM images of FNP and HFNP after treatment with H_2_O_2_. **Fig. S9.** DLS curves of FNP and HFNP after treatment with H_2_O_2_. **Fig. S10.** pH values and generated H_2_O_2_ concentrations at various time points arisen from GOX and HFNP@GOX@PFC catalyzed disintegration reaction of glucose. **Fig. S11.** Biostability of HFNP@GOX@PFC in gluconic acid with or without H_2_O_2_. **Fig. S12.** Intracellular ROS fluorescence intensity of 4T1 cells. **Fig. S13.** Corresponding quantitative MFI analysis of CD47. **Fig. S14.** Bcl-2, BAX, CytC and Casp3 relative mRNA expressions. **Fig. S15.** Gate strategy. **Fig. S16.** Wound healing rate of 4T1 cells. **Fig. S17. **Migration rate of 4T1 cells. **Fig. S18.** Invasion rate of 4T1 cells. **Fig. S19.** Body weight of tumor-bearing mice. **Fig. S20.** The quantitative IFC intensity analysis of CD47.** Fig. S21.** The quantitative IFC intensity analysis of CD86.** Fig. S22.** Biomedical blood index of tumor-bearing BALB/c mice. **Fig. S23.** H&E staining for major tissues. **Table S1.** Primer sequences.

## Data Availability

All data generated and analyzed during this research are included in this published article and additional file.
