# Supplementary material for: Cascade amplification of tumor chemodynamic therapy and starvation with re-educated TAMs via Fe-MOF based functional nanosystem
Source: J Nanobiotechnology. 2023 Apr 11;21:127. doi: 10.1186/s12951-023-01878-3 (PMC10088258; doi:10.1186/s12951-023-01878-3)
Supplement: Supplementary file 1 — Additional file 1: Fig. S1. Surface Zeta potential of FNP and HFNP. Fig. S2. XRD of FNP and HFNP. Fig. S3. H-NMR of FNP and HFNP. Fig. S4. FTIR of FNP and HFNP. Fig. S5. Linear relationships between the UV-vis absorbance and the concentration of GOX. Fig. S6. Efflux time of pure PFC and HFNP@GOX@PFC nanoparticles. Fig. S7. Linear relationships between the GC intensity and the concentration of PFC. Fig. S8. TEM images of FNP and HFNP after treatment with H2O2. Fig. S9. DLS curves of FNP and HFNP after treatment with H2O2. Fig. S10. pH values and generated H2O2 concentrations at various time points arisen from GOX and HFNP@GOX@PFC catalyzed disintegration reaction of glucose. Fig. S11. Biostability of HFNP@GOX@PFC in gluconic acid with or without H2O2. Fig. S12. Intracellular ROS fluorescence intensity of 4T1 cells. Fig. S13. Corresponding quantitative MFI analysis of CD47. Fig. S14. Bcl-2, BAX, CytC and Casp3 relative mRNA expressions. Fig. S15. Gate strategy. Fig. S16. Wound healing rate of 4T1 cells. Fig. S17. Migration rate of 4T1 cells. Fig. S18. Invasion rate of 4T1 cells. Fig. S19. Body weight of tumor-bearing mice. Fig. S20. The quantitative IFC intensity analysis of CD47. Fig. S21. The quantitative IFC intensity analysis of CD86. Fig. S22. Biomedical blood index of tumor-bearing BALB/c mice. Fig. S23. H&E staining for major tissues. Table S1. Primer sequences. [file 12951_2023_1878_MOESM1_ESM.docx]

Additional file information for

**Cascade amplification of tumor starvation and chemodynamic therapy via Fe-MOF based functional nanosystem**

Xinmin Zheng^1^, Xiang Li^1^, Siyu Meng^2^, Guolin Shi^1^, Hui Li^1^, Huiping Du^2^, Liangliang Dai^2*^ and Hui Yang^1*^

^1^ School of Life Sciences, Northwestern Polytechnical University, Xi’an 710072, China

^2^ Institute of Medical Research, Northwestern Polytechnical University, Xi’an 710072, China

*Corresponding author: [liangliangdai@nwpu.edu.cn](mailto:liangliangdai@nwpu.edu.cn) (L.L. Dai)

*Corresponding author: [kittyyh@nwpu.edu.cn](mailto:kittyyh@nwpu.edu.cn) ( H. Yang)

***Lift of Contents***

**[Fig. S1](#_Toc127737065)** [Surface Zeta potential of FNP and HFNP S4](#_Toc127737065)

**[Fig. S2](#_Toc127737066)** [XRD of FNP and HFNP S5](#_Toc127737066)

**[Fig. S3](#_Toc127737067)** [H-NMR of FNP and HFNP S6](#_Toc127737067)

**[Fig. S4](#_Toc127737068)** [FTIR of FNP and HFNP S7](#_Toc127737068)

**[Fig. S5](#_Toc127737069)** [Linear relationships between the UV-vis absorbance and the concentration of GOX S8](#_Toc127737069)

**[Fig. S6](#_Toc127737070)** [Efflux time of pure PFC and HFNP@GOX@PFC nanoparticles S9](#_Toc127737070)

**[Fig. S7](#_Toc127737071)** [Linear relationships between the GC intensity and the concentration of PFC S10](#_Toc127737071)

**[Fig. S8](#_Toc127737072)** [TEM images of FNP and HFNP after treatment with H](#_Toc127737072)_[2](#_Toc127737072)_[O](#_Toc127737072)_[2](#_Toc127737072)_ [S11](#_Toc127737072)

**[Fig. S9](#_Toc127737073)** [DLS curves of FNP and HFNP after treatment with H](#_Toc127737073)_[2](#_Toc127737073)_[O](#_Toc127737073)_[2](#_Toc127737073)_ [S12](#_Toc127737073)

**[Fig. S10](#_Toc127737073)** [pH values and generated H](#_Toc127737073)_[2](#_Toc127737073)_[O](#_Toc127737073)_[2](#_Toc127737073)_ [concentrations at various time points arisen from GOX and HFNP@GOX@PFC catalyzed disintegration reaction of glucose S13](#_Toc127737073)

**[Fig. S11](#_Toc127737074)** [Biostability of HFNP@GOX@PFC in gluconic acid with or without H](#_Toc127737074)_[2](#_Toc127737074)_[O](#_Toc127737074)_[2](#_Toc127737074)_ [S14](#_Toc127737074)

**[Fig. S12](#_Toc127737075)** [Intracellular ROS ﬂuorescence intensity of 4T1 cells S15](#_Toc127737075)

**[Fig. S13](#_Toc127737076)** [Corresponding quantitative MFI analysis of CD47 S16](#_Toc127737076)

**[Fig. S14](#_Toc127737077)** [Bcl-2, BAX, CytC and Casp3 relative mRNA expressions S17](#_Toc127737077)

**[Fig. S15](#_Toc127737078)** [Gate strategy S18](#_Toc127737078)

**[Fig. S16](#_Toc127737079)** [Wound healing rate of 4T1 cells S19](#_Toc127737079)

**[Fig. S17](#_Toc127737080)** [Migration rate of 4T1 cells S20](#_Toc127737080)

**[Fig. S18](#_Toc127737081)** [Invasion rate of 4T1 cells S21](#_Toc127737081)

**[Fig. S19](#_Toc127737082)** [Body weight of tumor-bearing mice S22](#_Toc127737082)

**[Fig. S20](#_Toc127737083)** [The quantitative IFC intensity analysis of CD47 S23](#_Toc127737083)

**[Fig. S21](#_Toc127737084)** [The quantitative IFC intensity analysis of CD86 S24](#_Toc127737084)

**[Fig. S22](#_Toc127737085)** [Biomedical blood index of tumor-bearing BALB/c mice S25](#_Toc127737085)

**[Fig. S23](#_Toc127737086)** [H&E staining for major tissues S26](#_Toc127737086)

**[Table S1.](#_Toc127737087)** [Primer sequences S27](#_Toc127737087)


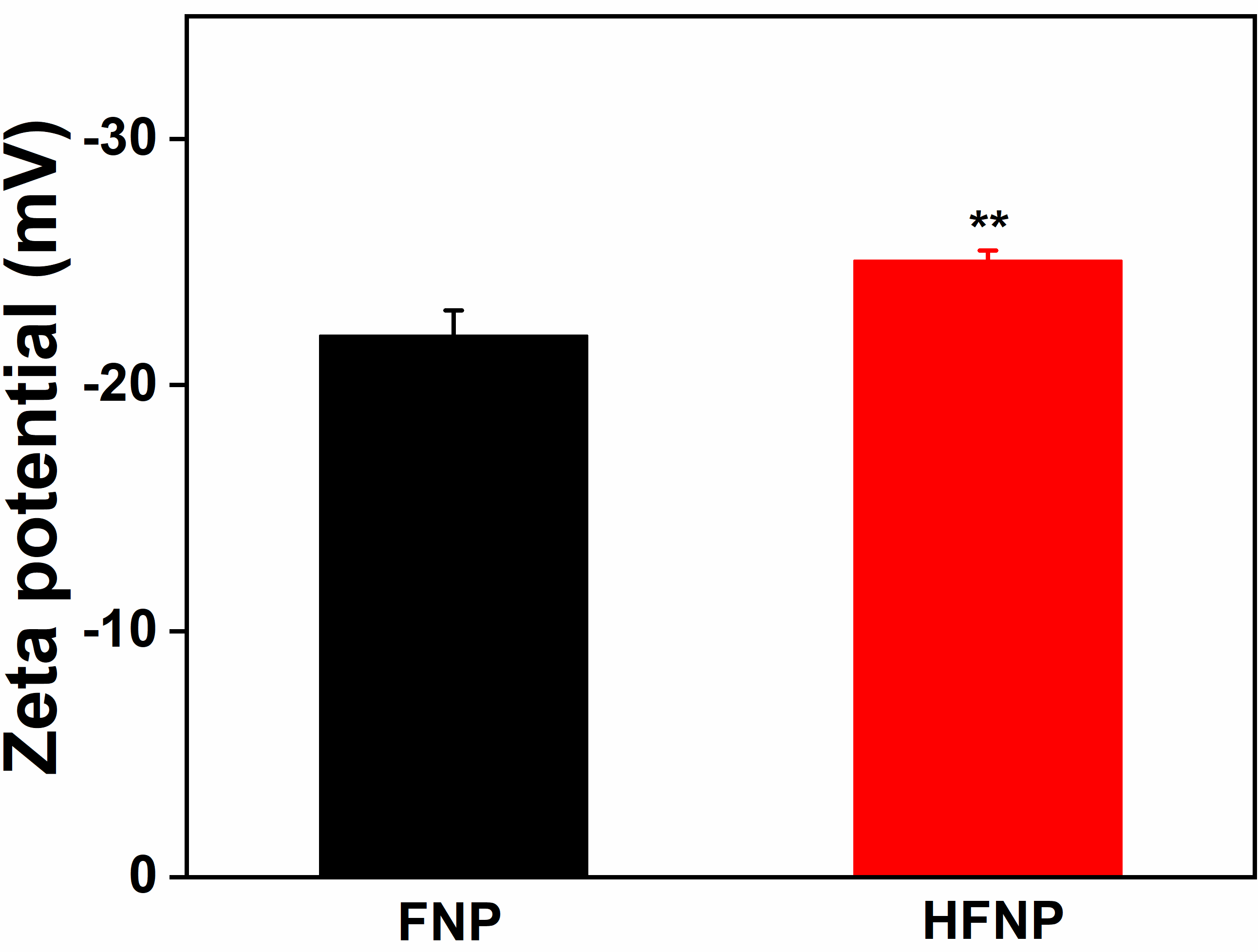


**Fig. S1** Surface Zeta potential of FNP and HFNP measured using DLS. Error bars present as mean ± SD (n = 6).


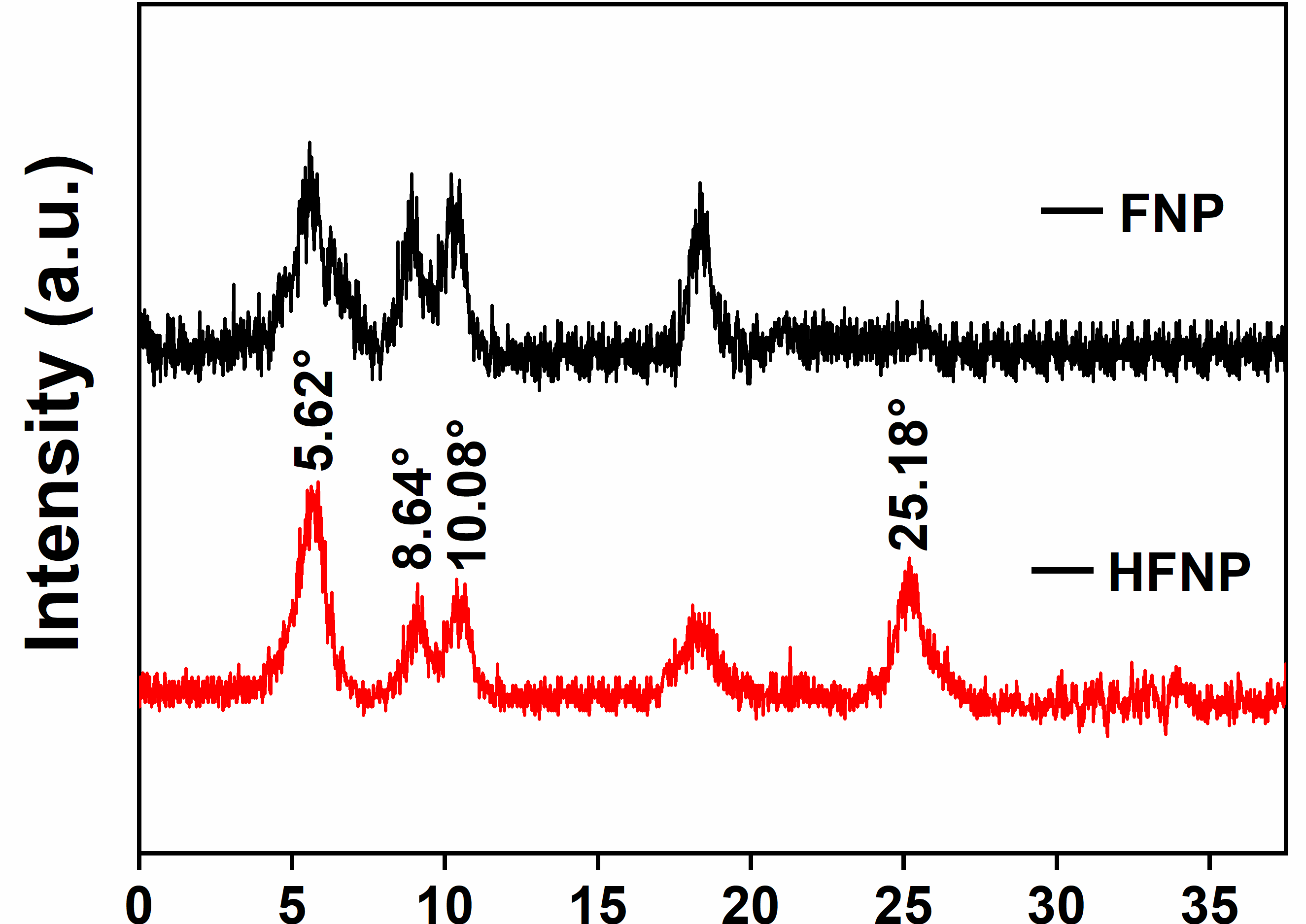


**Fig. S2** XRD of FNP and HFNP.


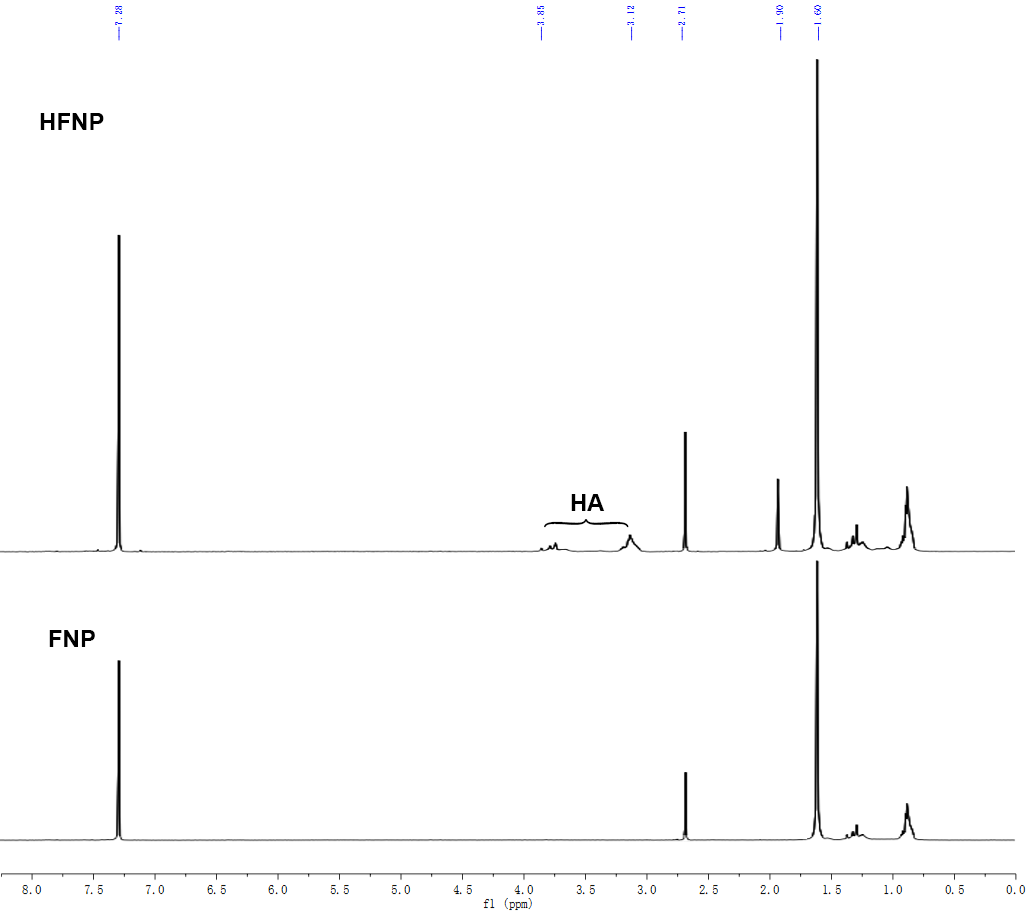


**Fig. S3** H-NMR of FNP and HFNP.

**

**

**Fig. S4** FTIR of FNP and HFNP.


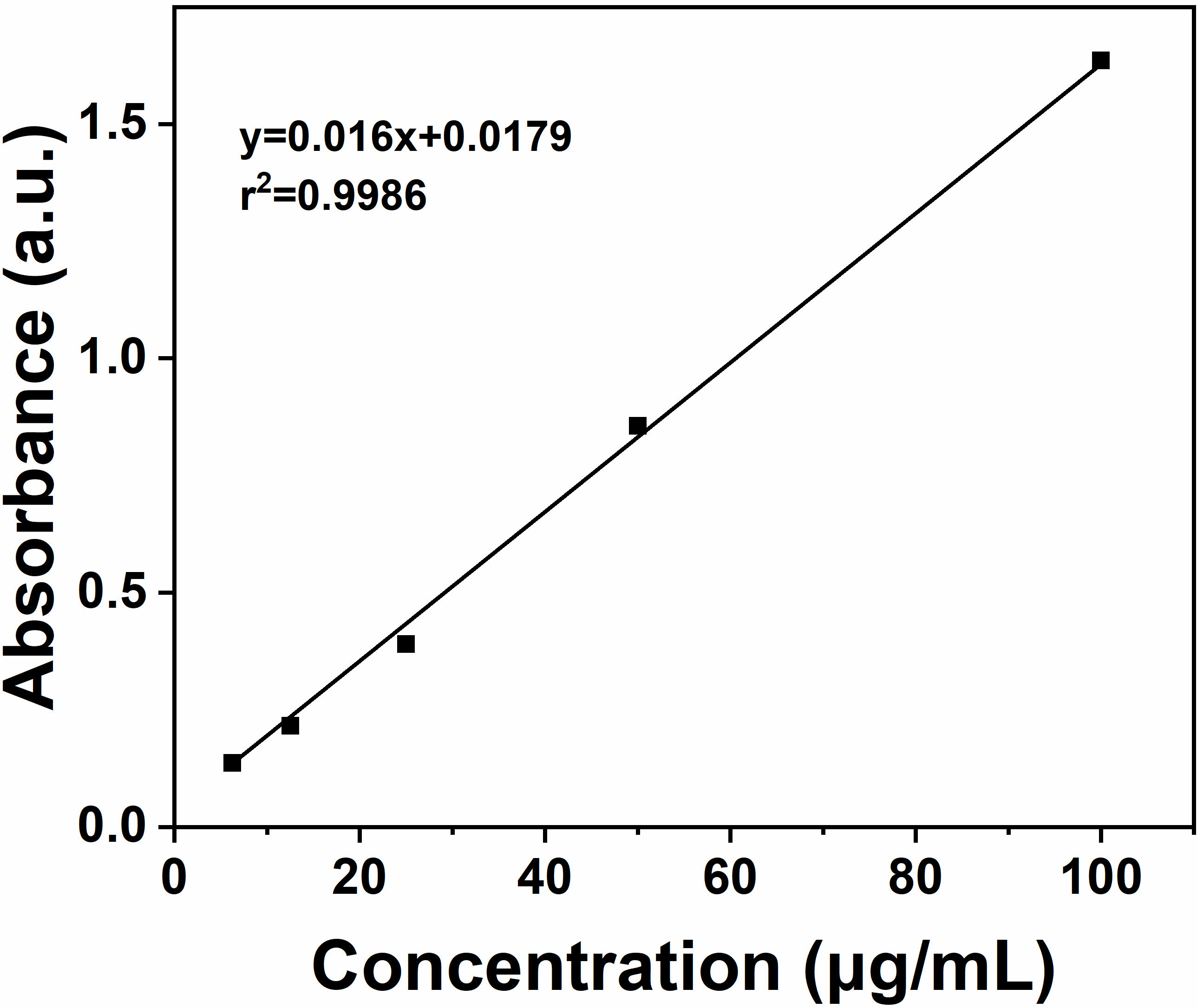


**Fig. S5** Linear relationships between the UV-vis absorbance and the concentration of GOX.


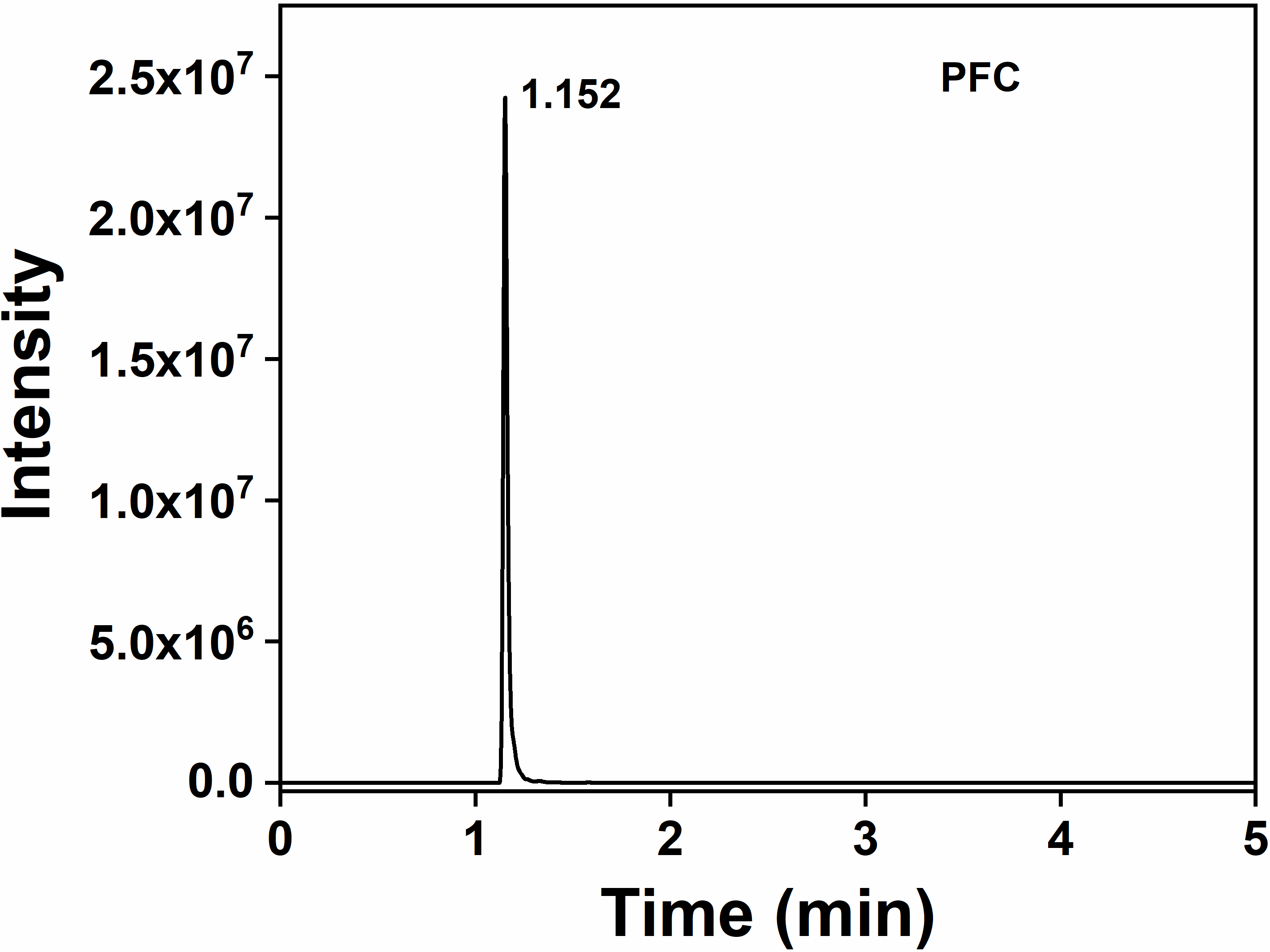

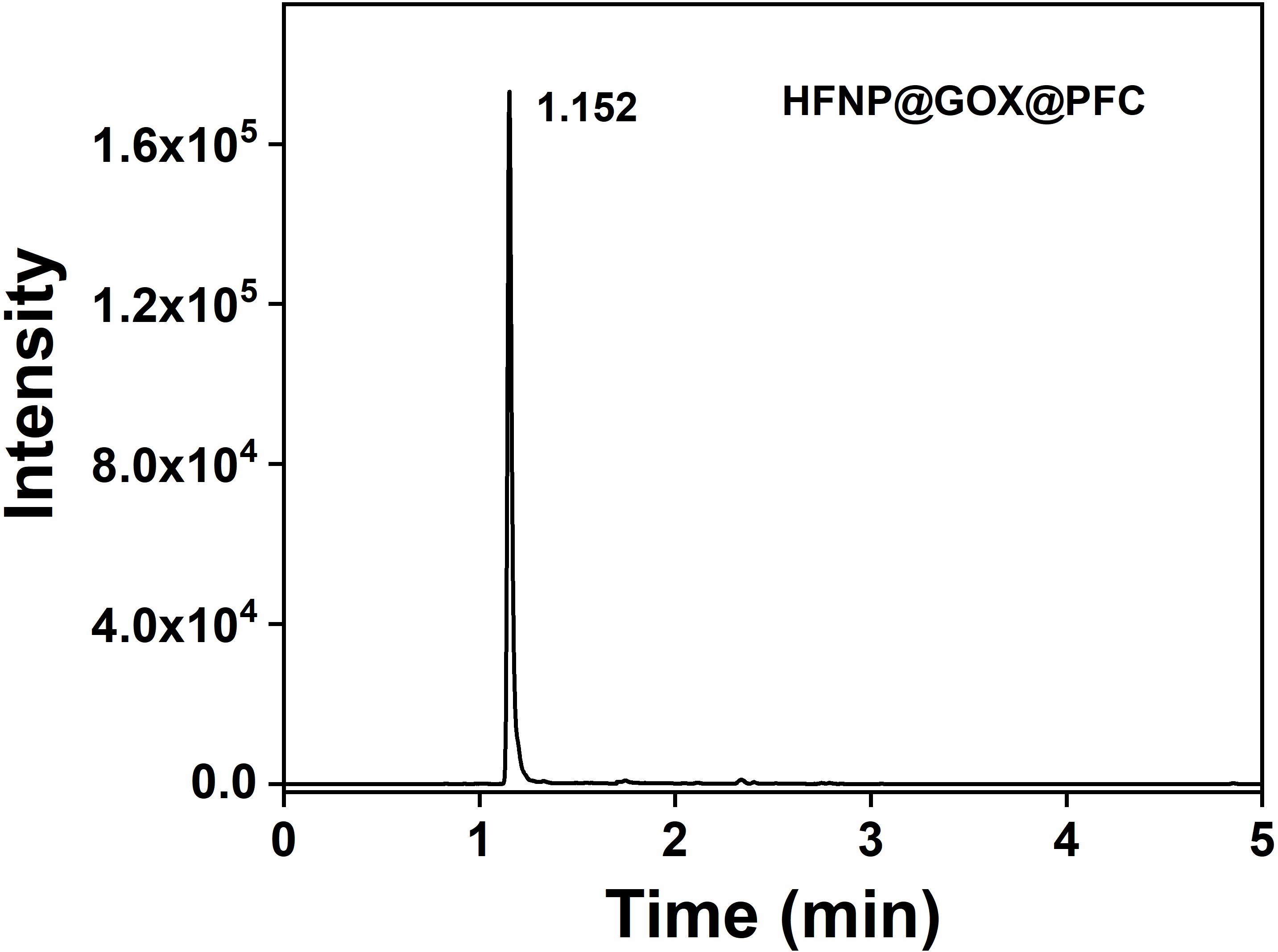


(b)

(a)

**Fig. S6** Efflux time of (a) pure PFC and (b) HFNP@GOX@PFC nanoparticles measured by GC. The samples were extracted by acetonitrile and pentafluorobutane before GC-MS measurement following a literature protocol.


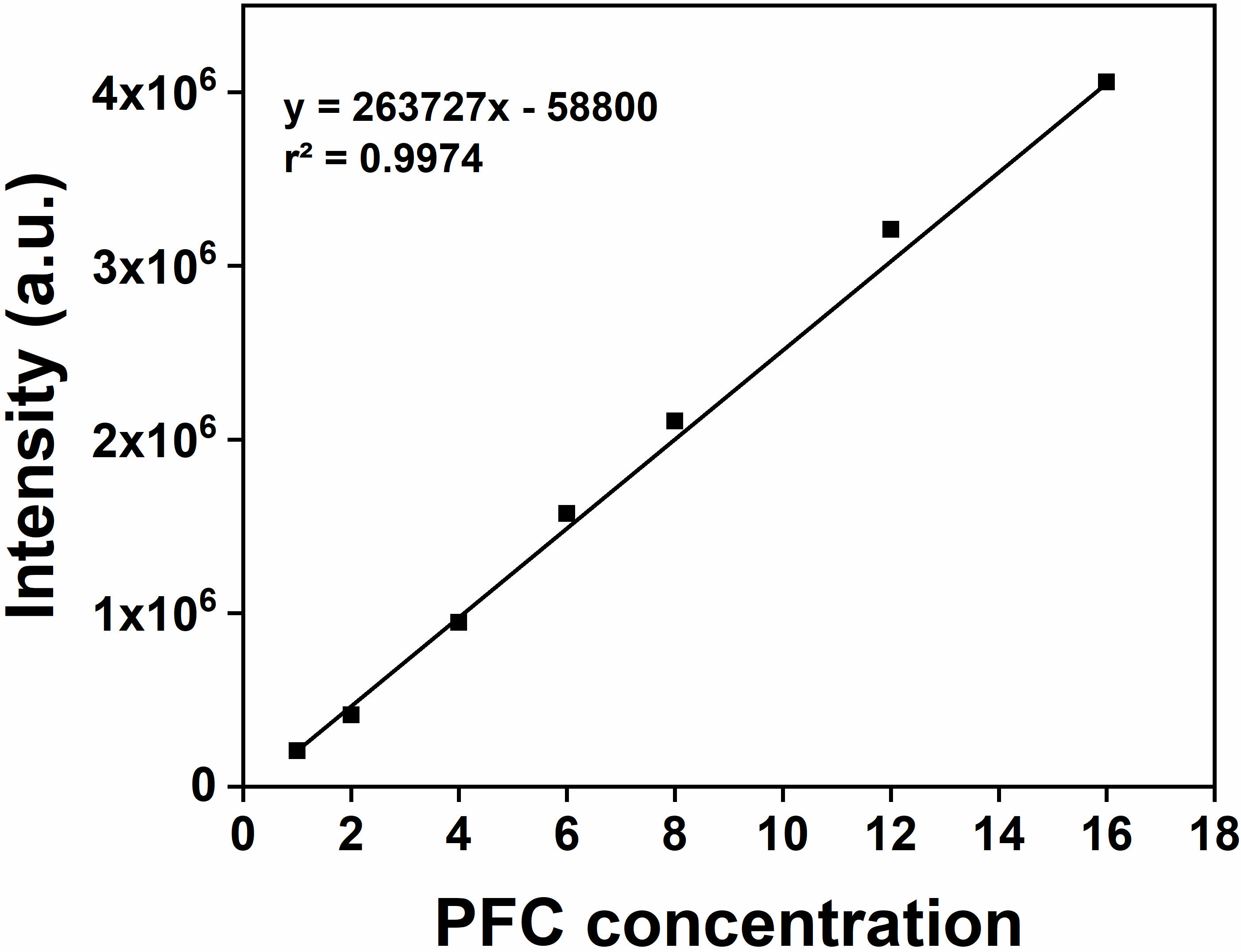


**Fig. S7** Linear relationships between the GC intensity and the concentration of PFC.


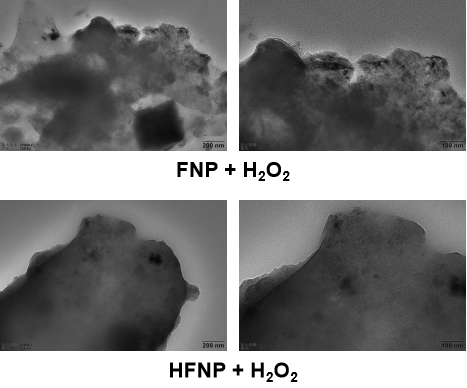


**Fig. S8** TEM images of FNP and HFNP after treatment with H_2_O_2_. Scale bar: 200 nm for left figures and 100 nm for right figures.





**Fig. S9** DLS curves of FNP and HFNP after treatment with H_2_O_2_.







(b)

(a)

**Fig. S10** pH values and generated H_2_O_2_ concentrations at various time points arisen from (a) GOX and (b) HFNP@GOX@PFC catalyzed disintegration reaction of glucose. Error bars present as mean ± SD (n = 3).





**Fig. S11** Biostability of HFNP@GOX@PFC in gluconic acid with or without H_2_O_2_. Error bars present as mean ± SD (n = 3).


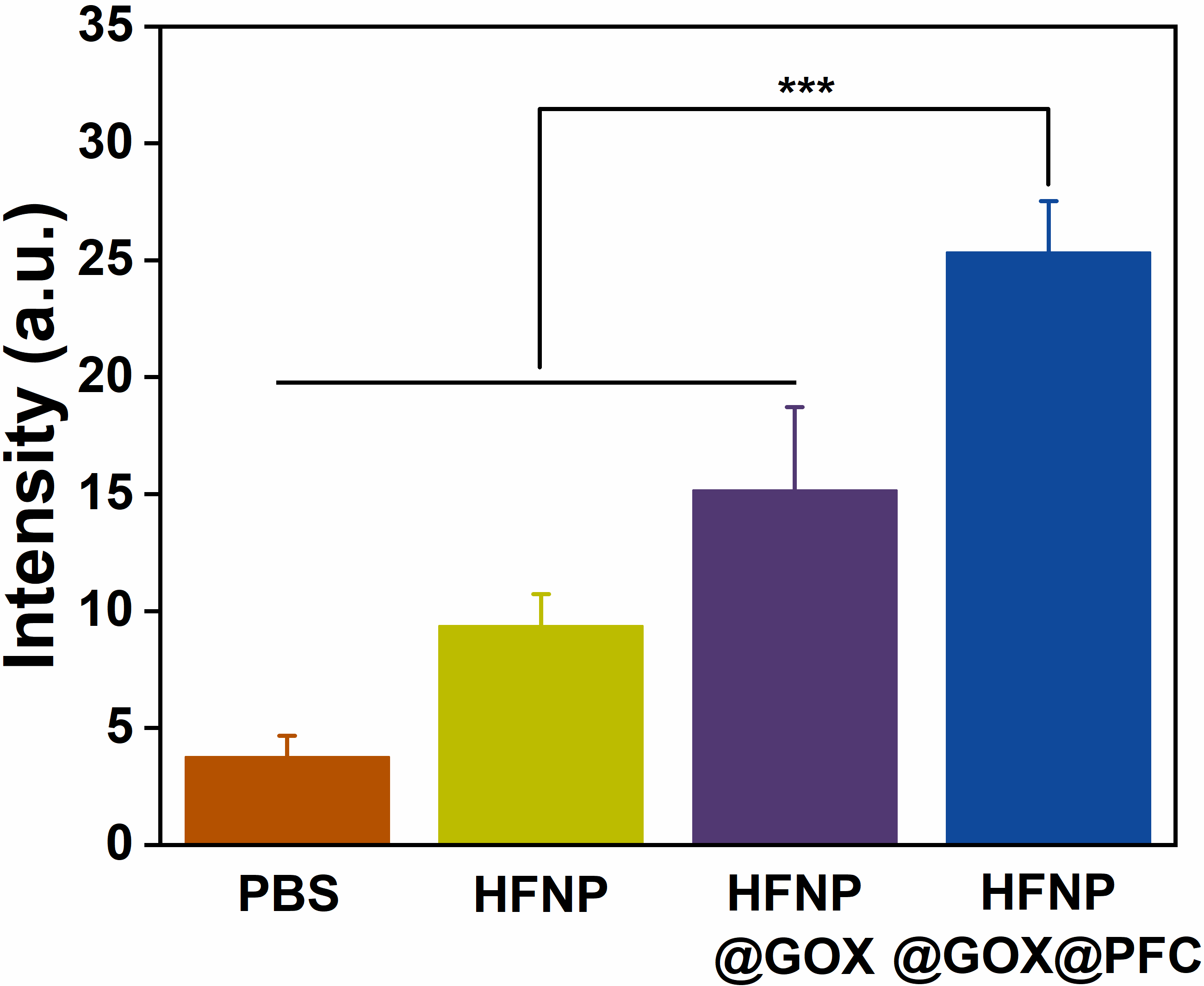


**Fig. S12** Intracellular ROS ﬂuorescence intensity of 4T1 cells. Error bars present as mean ± SD (n = 6).


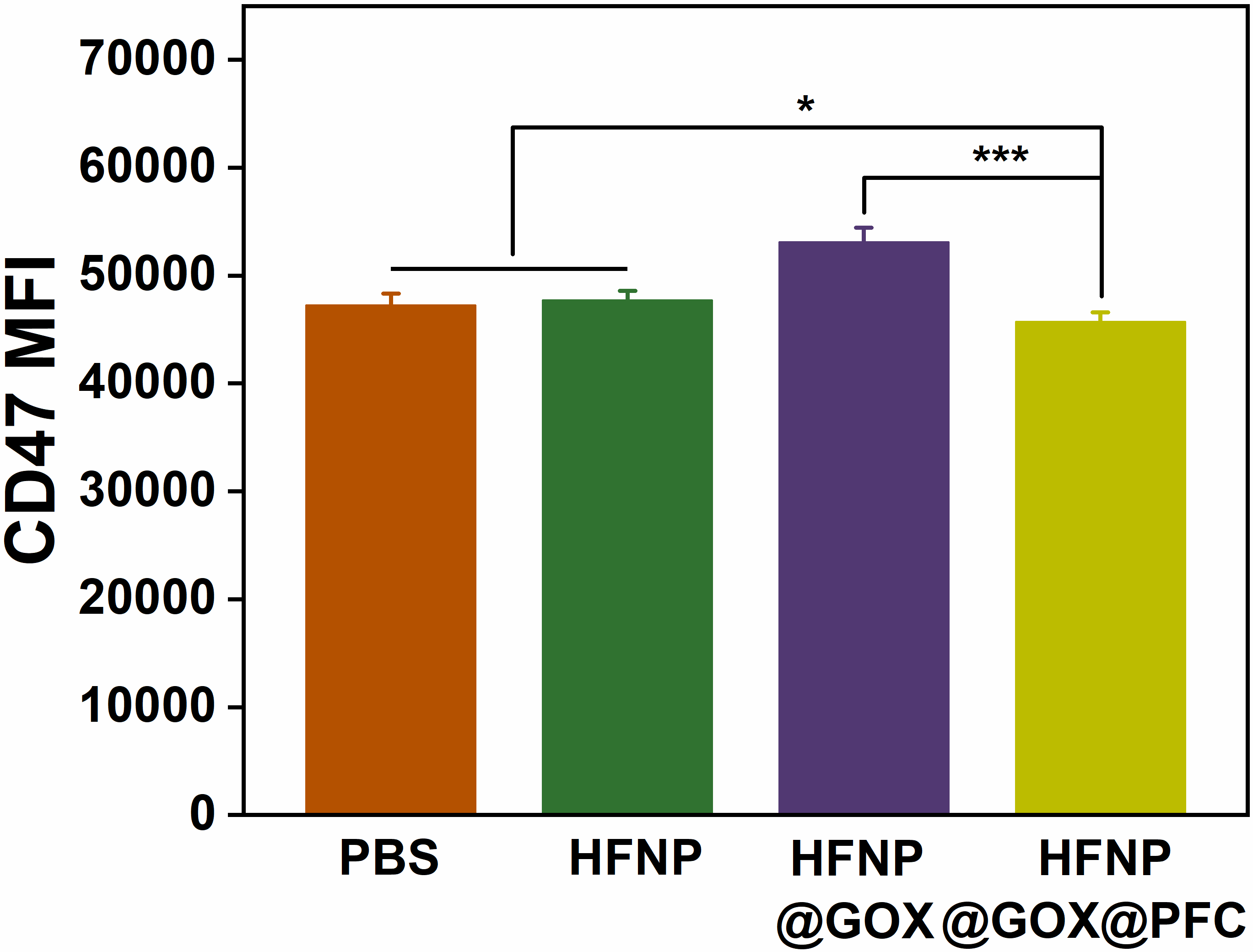


**Fig. S13** Corresponding quantitative MFI analysis of CD47 expression in 4T1 cells after treatment with PBS, HFNP, HFNP@GOX & HFNP@GOX@PFC for 24 h based on Fig. 4D. Error bars present as mean ± SD (n = 6).

**
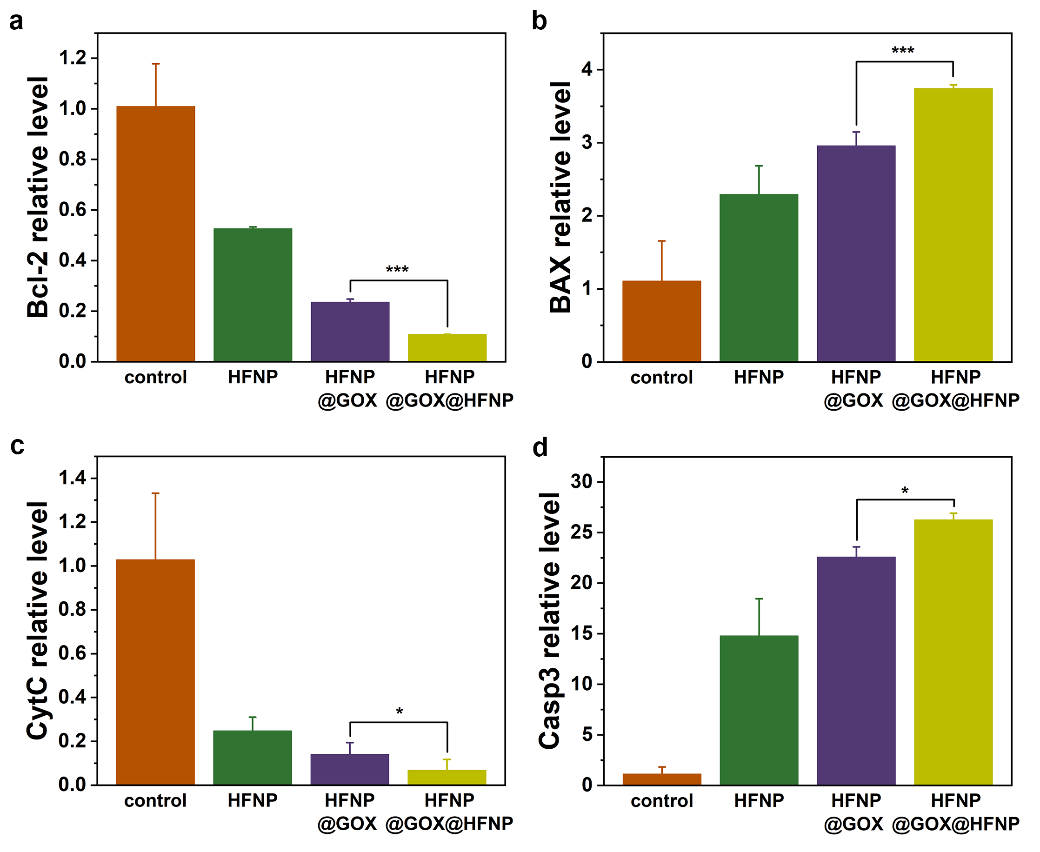
**

**Fig. S14** (a) Bcl-2, (b) BAX, (c) CytC and (d) Casp3 relative mRNA expressions analysis of 4T1 cells treated with PBS, HFNP & HFNP@GOX@PFC. Error bars present as mean ± SD (n = 6).


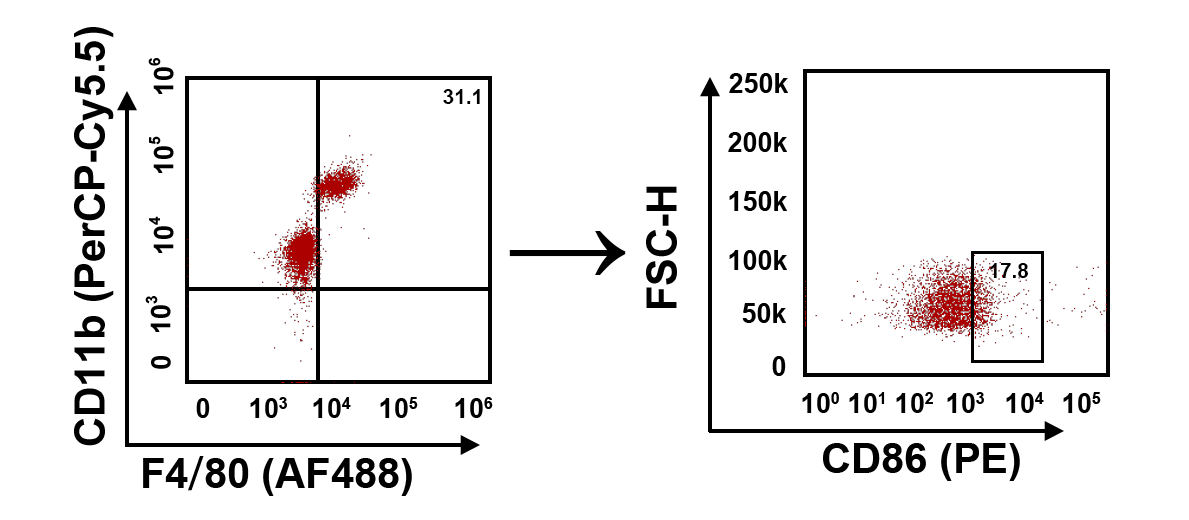


**Fig. S15** Gate strategy for assessing TAMs polarization by following the indicated treatments.


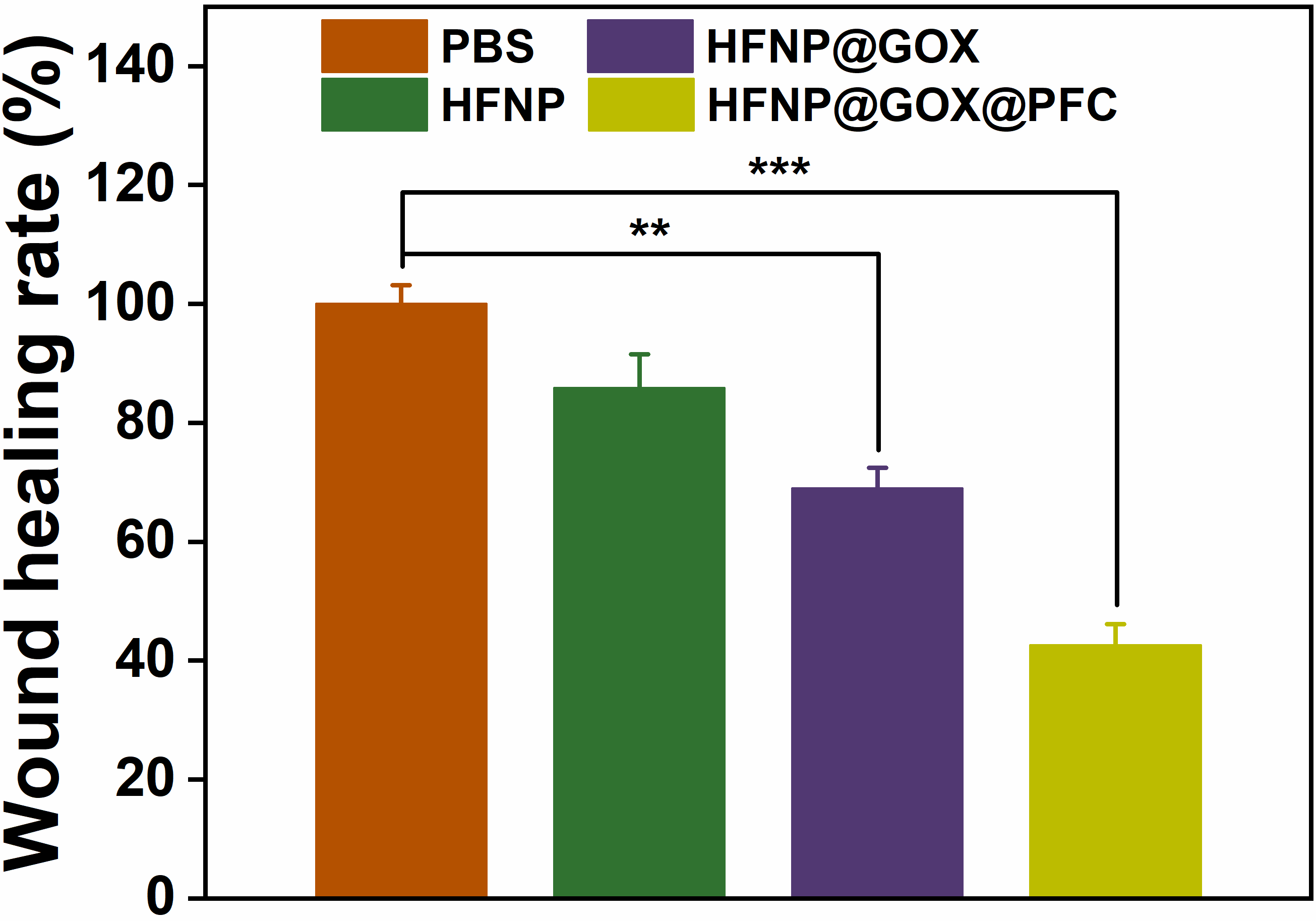


**Fig. S16** Wound healing rate of 4T1 cells after various treatments. Error bars present as mean ± SD (n = 6).


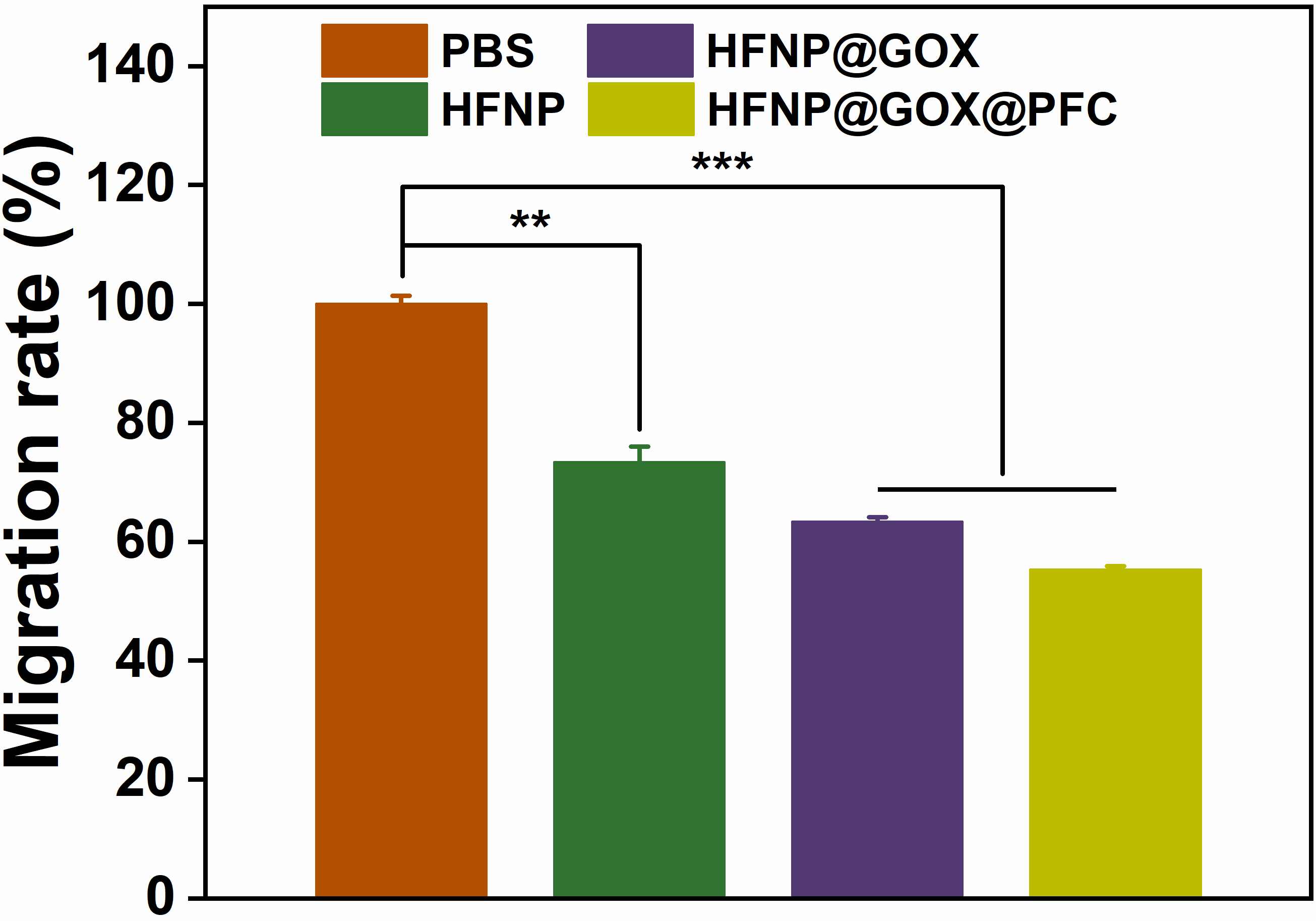


**Fig. S17** Migration rate of 4T1 cells after various treatments. Error bars present as mean ± SD (n = 6).


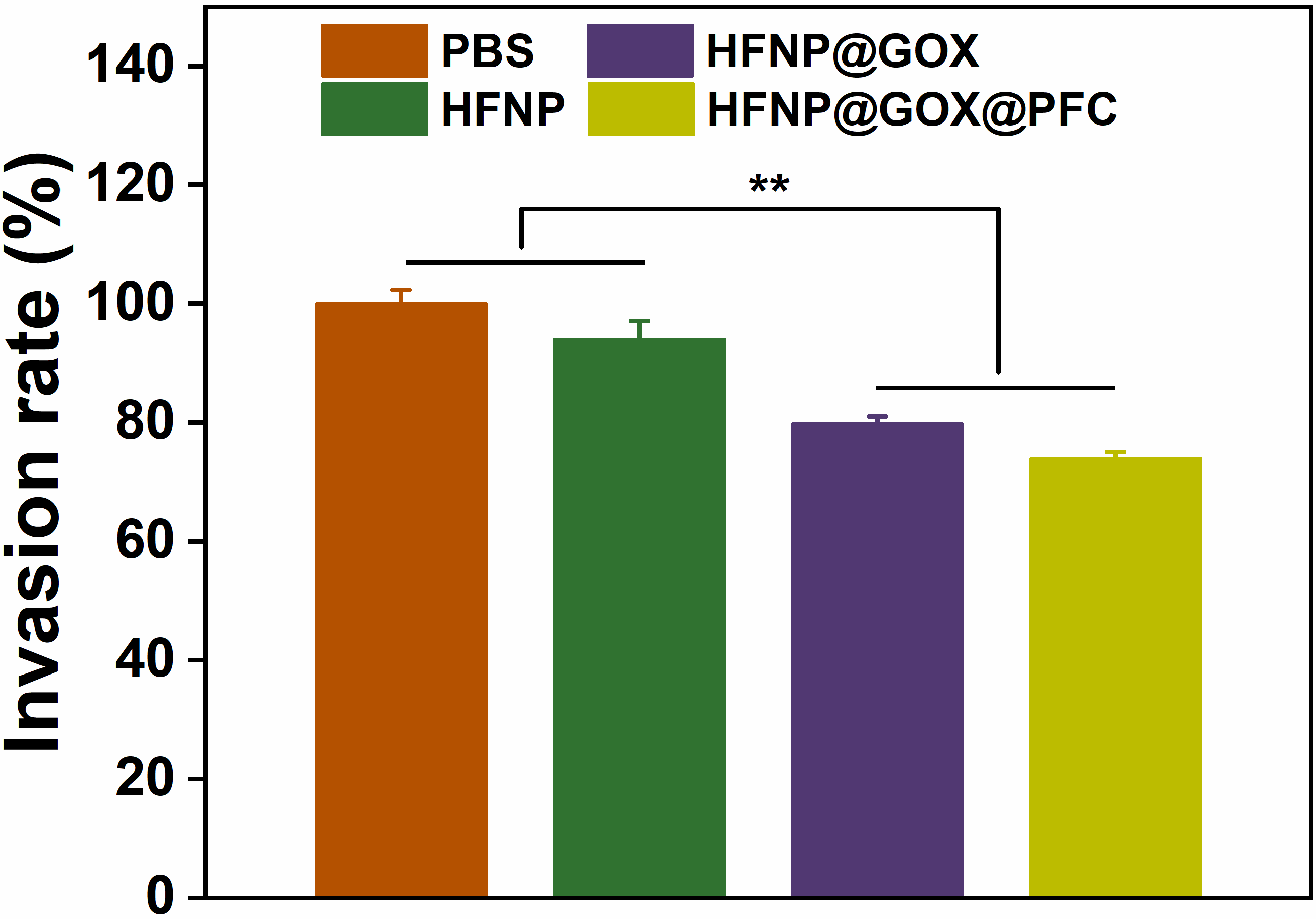


**Fig. S18** Invasion rate of 4T1 cells after various treatments. Error bars present as mean ± SD (n = 6).


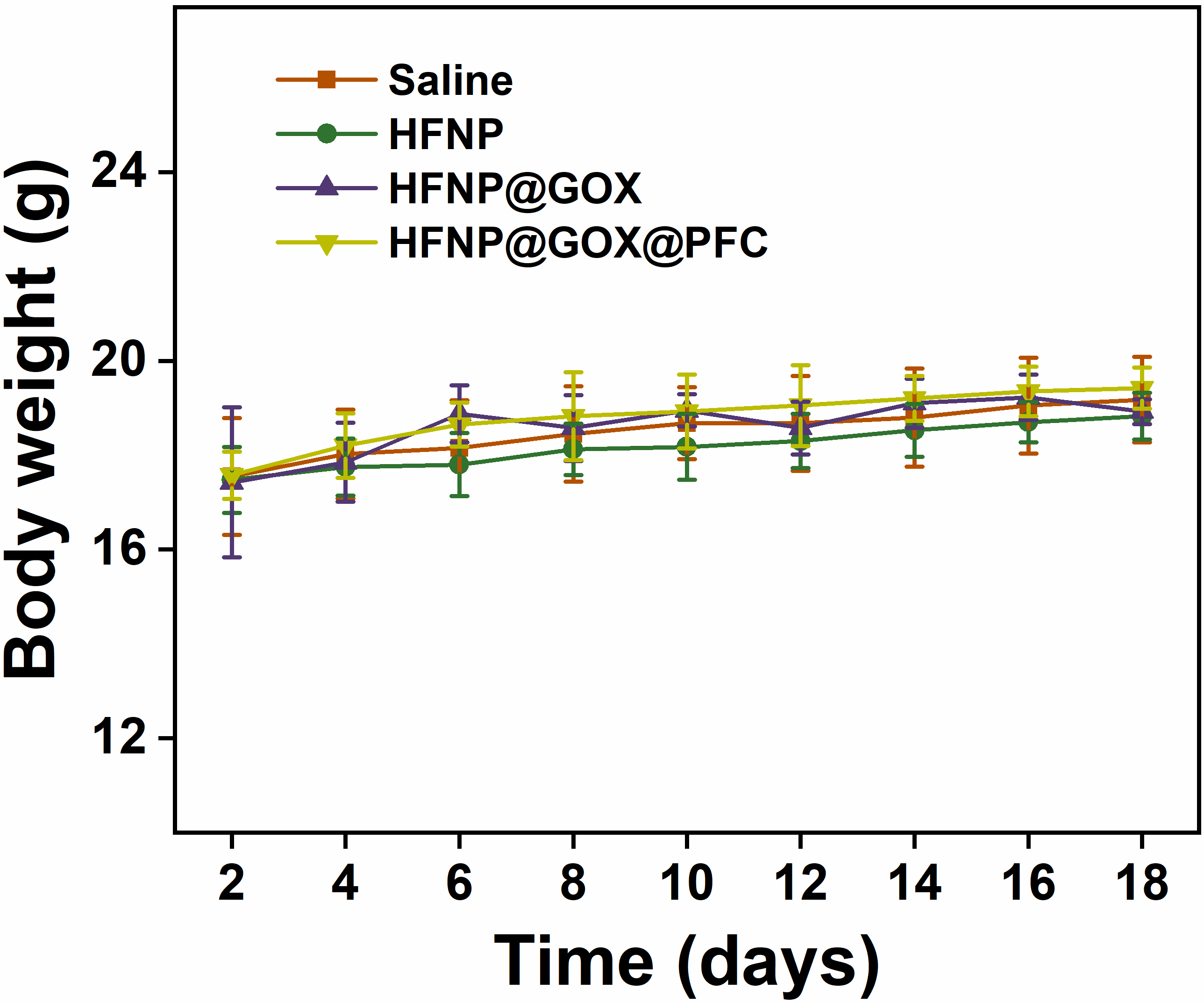


**Fig. S19** Body weight of tumor-bearing mice after various treatments for 18 days. Error bars present as mean ± SD (n = 6).


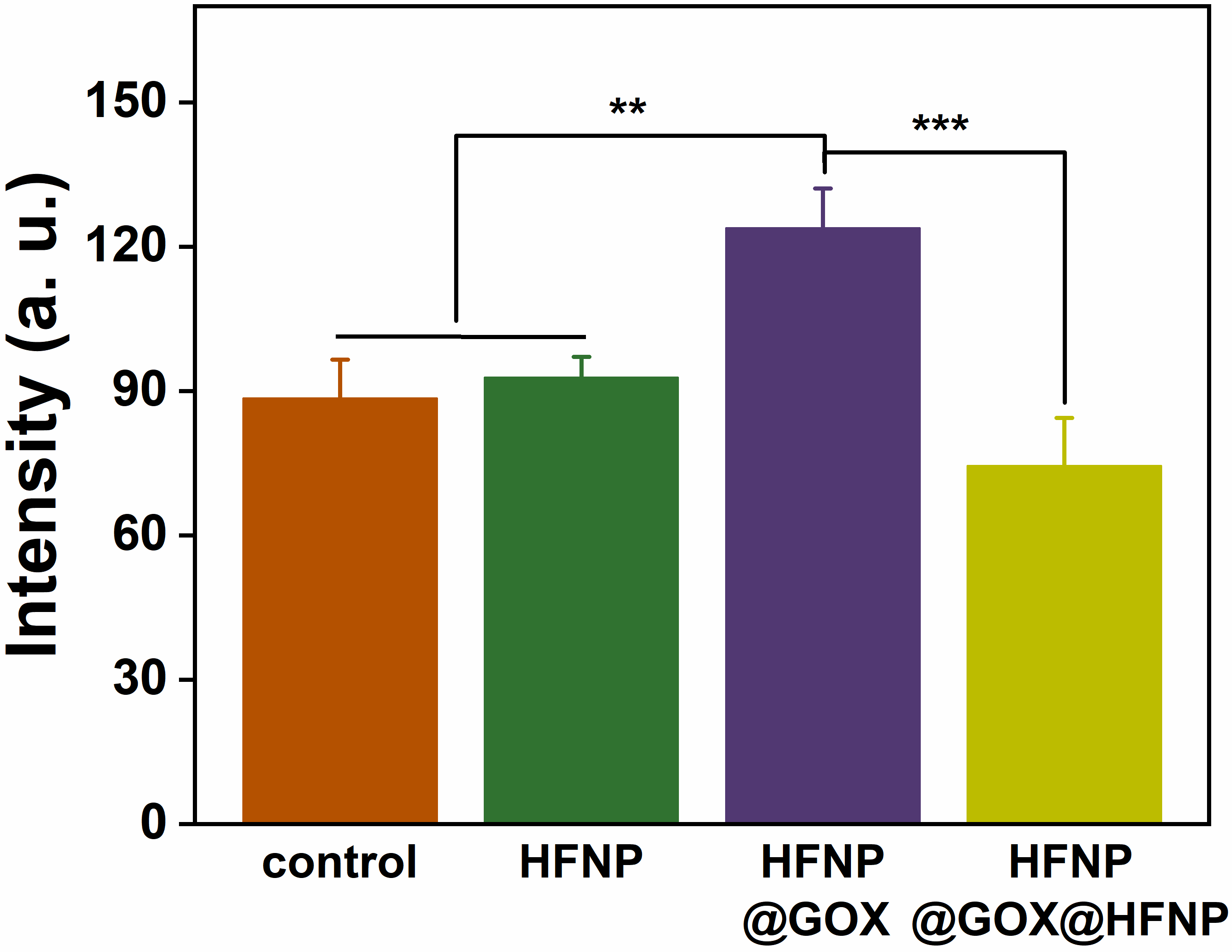


**Fig. S20** The quantitative IFC intensity analysis of CD47 based on Fig. 6D. Error bars present as mean ± SD (n = 6).


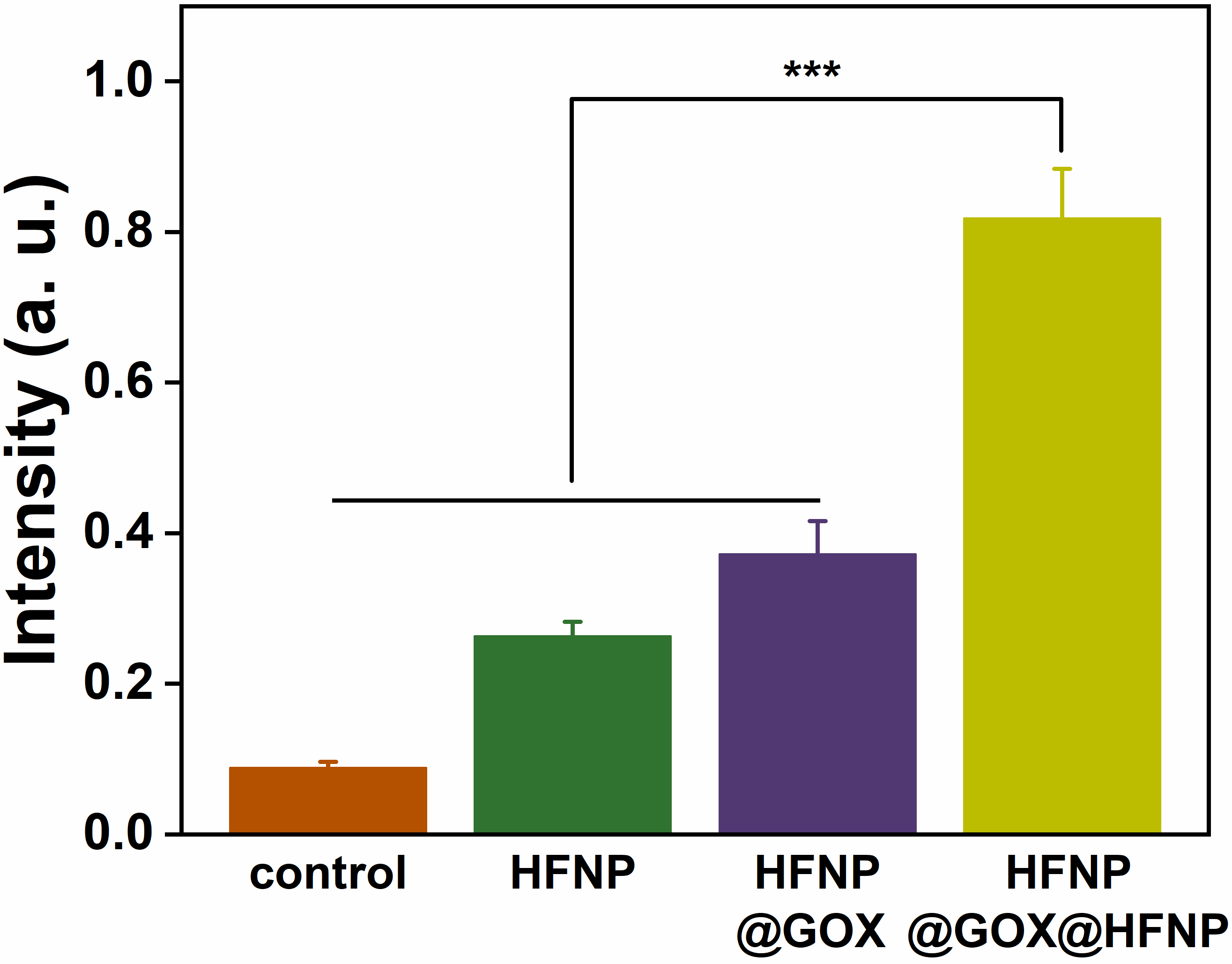


**Fig. S21** The quantitative IFC intensity analysis of CD86 based on Fig. 6D. Error bars present as mean ± SD (n = 6).


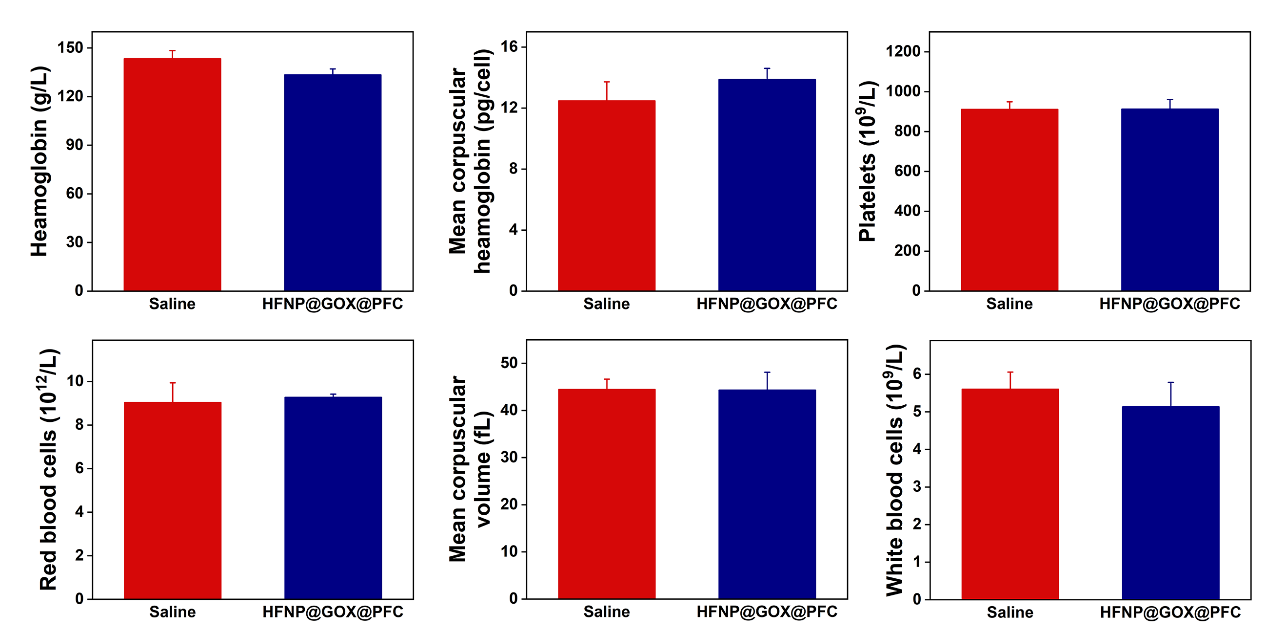


**Fig. S22** Biomedical blood index of tumor-bearing BALB/c mice after treatment with saline and HFNP@GOX@PFC for 18 days. Error bars present as mean ± SD (n = 4).


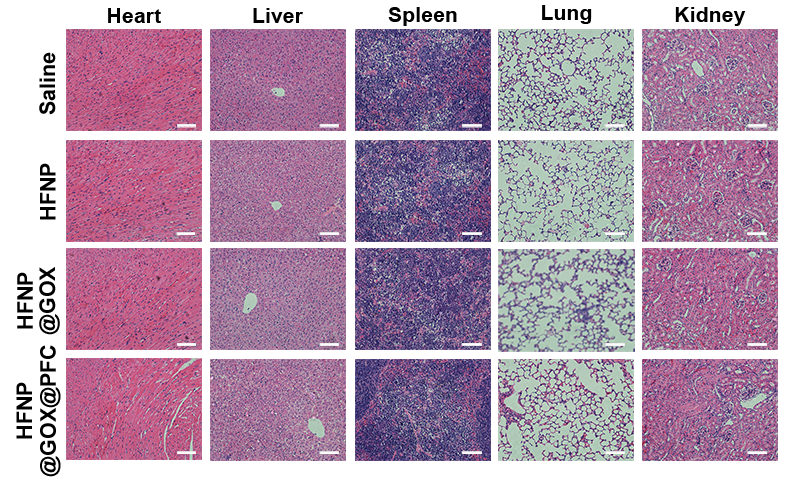


**Fig. S23** H&E staining for major tissues (heart, spleen, liver and kidney). Scale bar: 100 μm.

**Table S1.** Primer sequences.

| Gene | Forward Primer (5’→3’) | Reverse Primer (5’→3’) |
| --- | --- | --- |
| BCL-2 | ATCGCCCTGTGGATGACTGA | CAGAGACAGCCAGGAGAAATCA |
| BAX | TGCTAGCAAACTGGTGCTCA | GGTCCCGAAGTAGGAGAGGA |
| Casp3 | CATCTGCATCCGTCCTGA | CTCTCGGCTGTGGTGGGTGAA |
| CytC | CACCGACACCGGTACATAGG | TCTCCCCAGGTGATGCCTTT |
| β-actin | GGTGTGATGGTGGGAATGGG | ACGGTTGGCCTTAGGGTTCAG |
